# Supplementary figures and images for: Psittacosaurus houi, a longer snouted psittacosaurid from the Lower Cretaceous Lujiatun Unit of Yixian Formation, China, with the synonymy of the unresolved genus Hongshanosaurus revisited
Source: PeerJ. 2025 Jul 8;13:e19547. doi: 10.7717/peerj.19547 (PMC12248233; doi:10.7717/peerj.19547)

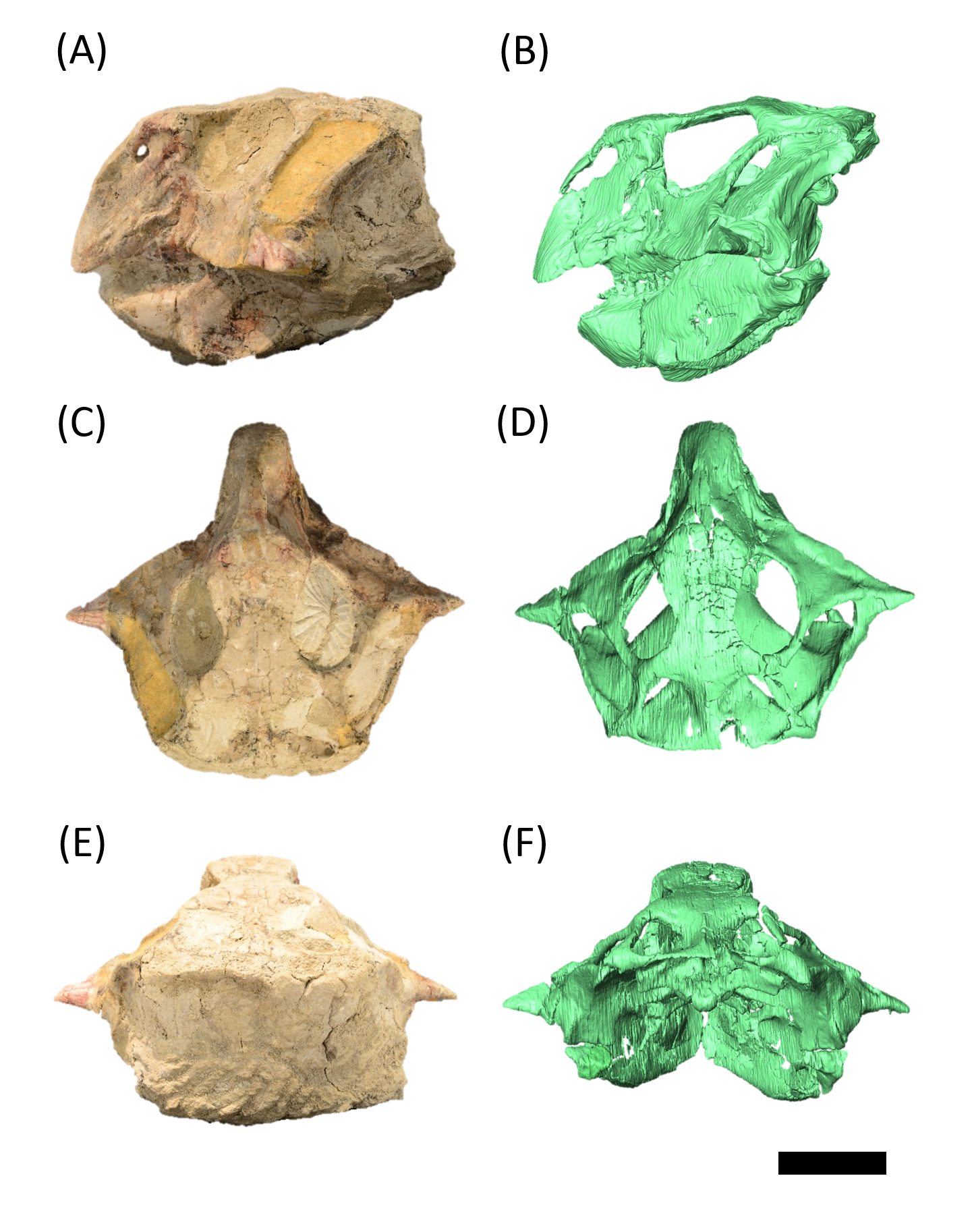

Supplement: Supplemental Information 1 — Scale bar equals 50 mm. [file peerj-13-19547-s001.png]

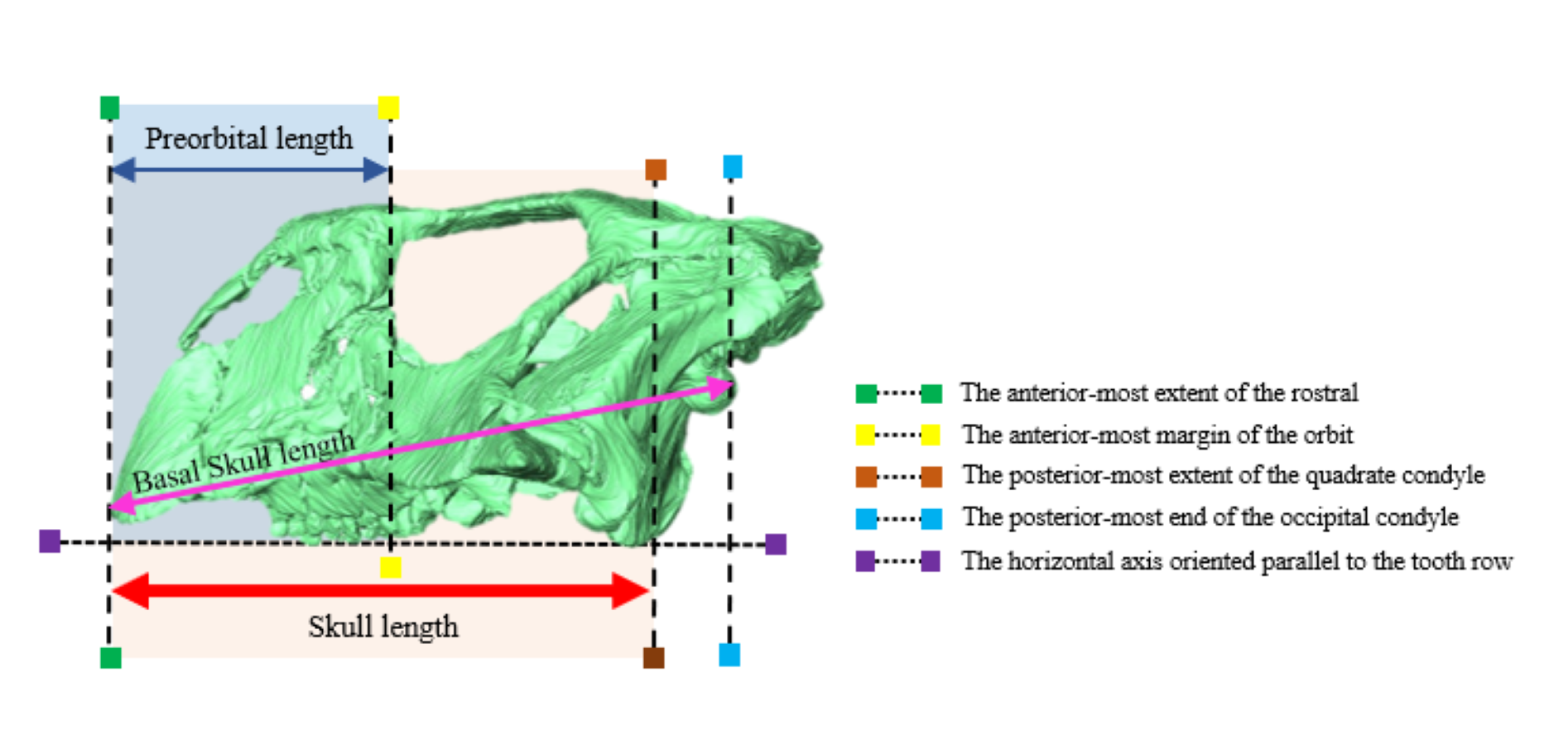

Supplement: Supplemental Information 2 [file peerj-13-19547-s002.png]

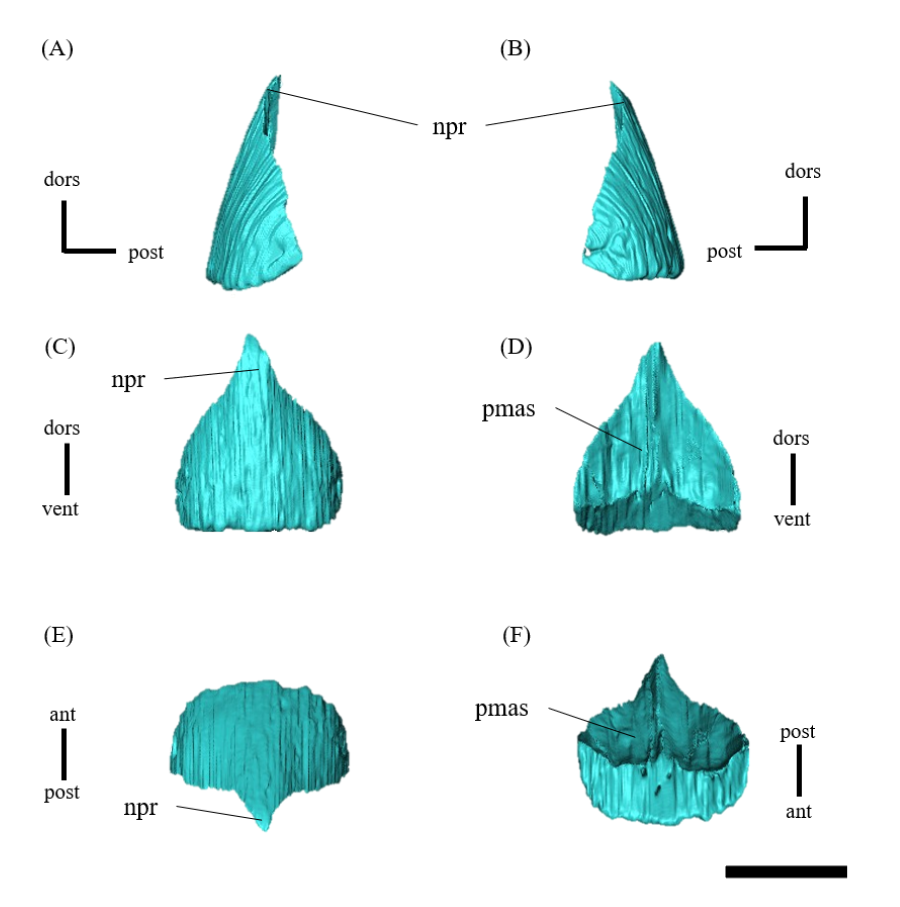

Supplement: Supplemental Information 3 — Abbreviations: npr, nasal process of rostral; pmas, premaxillary articular surface. Scale bar equals 20 mm. [file peerj-13-19547-s003.png]

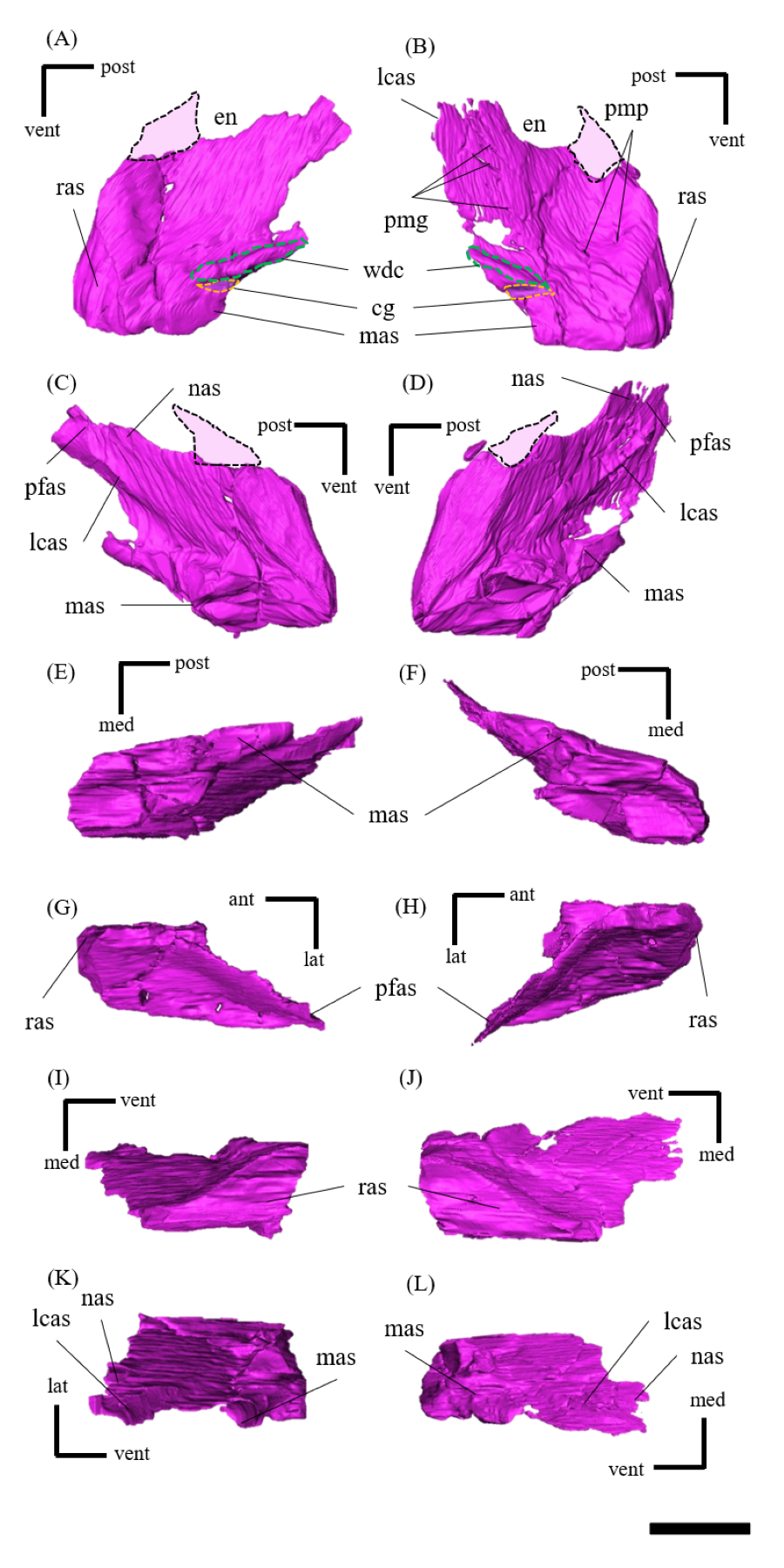

Supplement: Supplemental Information 4 — Green dotted line indicates well-developed crest, orange dotted line indicates conspicuous groove, and black dotted line indicates the missing part. Abbreviations: cg, conspicuous groove; en, external naris; lcas, lacrimal articular surface; mas, maxillary articular surface; nas, nasal articular surface; pfas, prefrontal articular surface; pmg, premaxillary groove; pmp, premaxillary pit; ras, rostral articular surface; wdc, well-developed crest. Scale bar equals 20 mm. [file peerj-13-19547-s004.png]

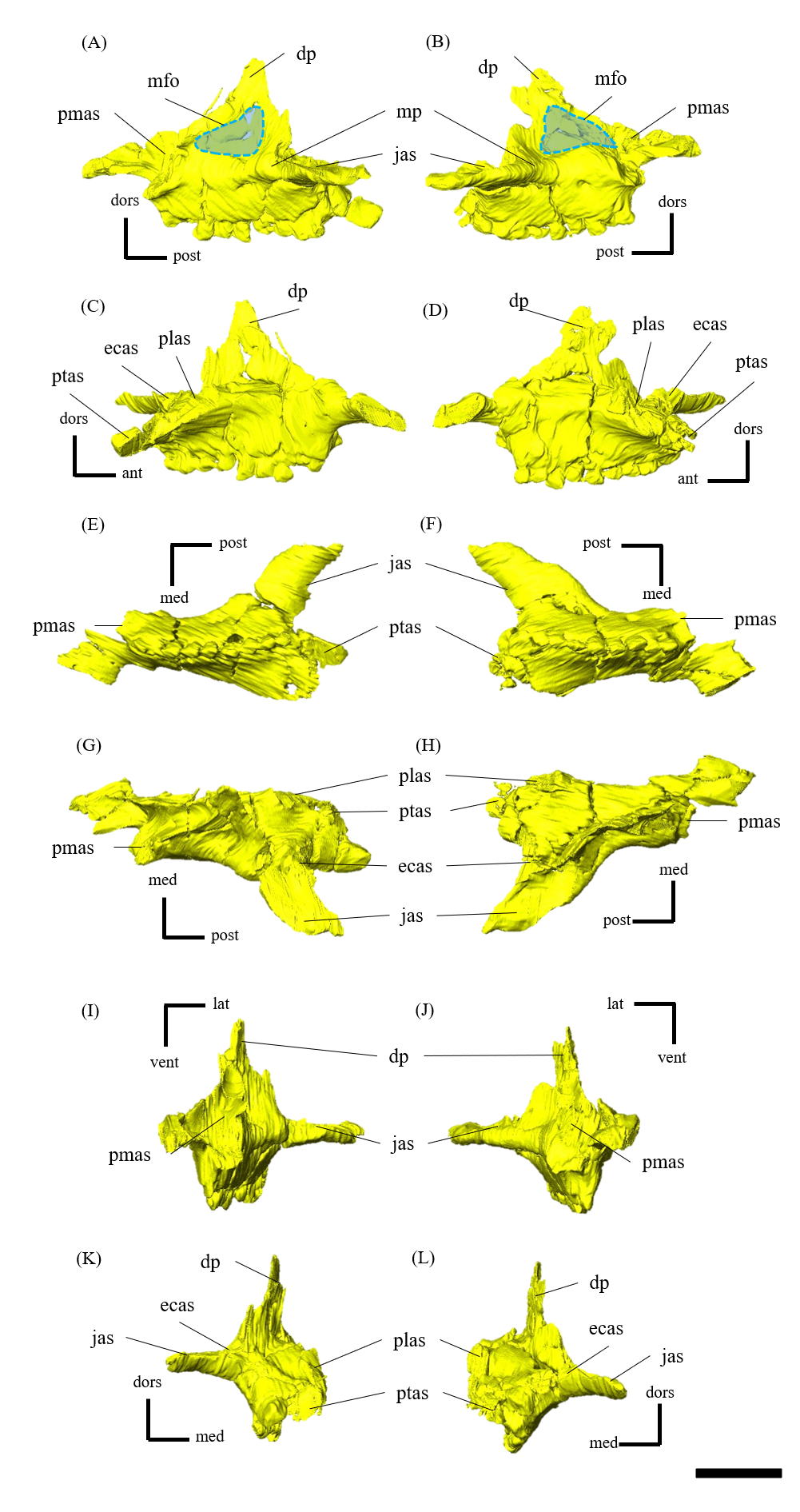

Supplement: Supplemental Information 5 — Blue dotted line indicates the position of maxillary fossa. Abbreviations: dp, dorsal process; ecas, ectopterygoid articular surface; jas, jugal articular surface; mfo, maxillary fossa; mp, maxillary protuberance; plas, palatine articular surface; pmas, premaxillary articular surface; ptas, pterygoid articular surface. Scale bar equals 20 mm. [file peerj-13-19547-s005.png]

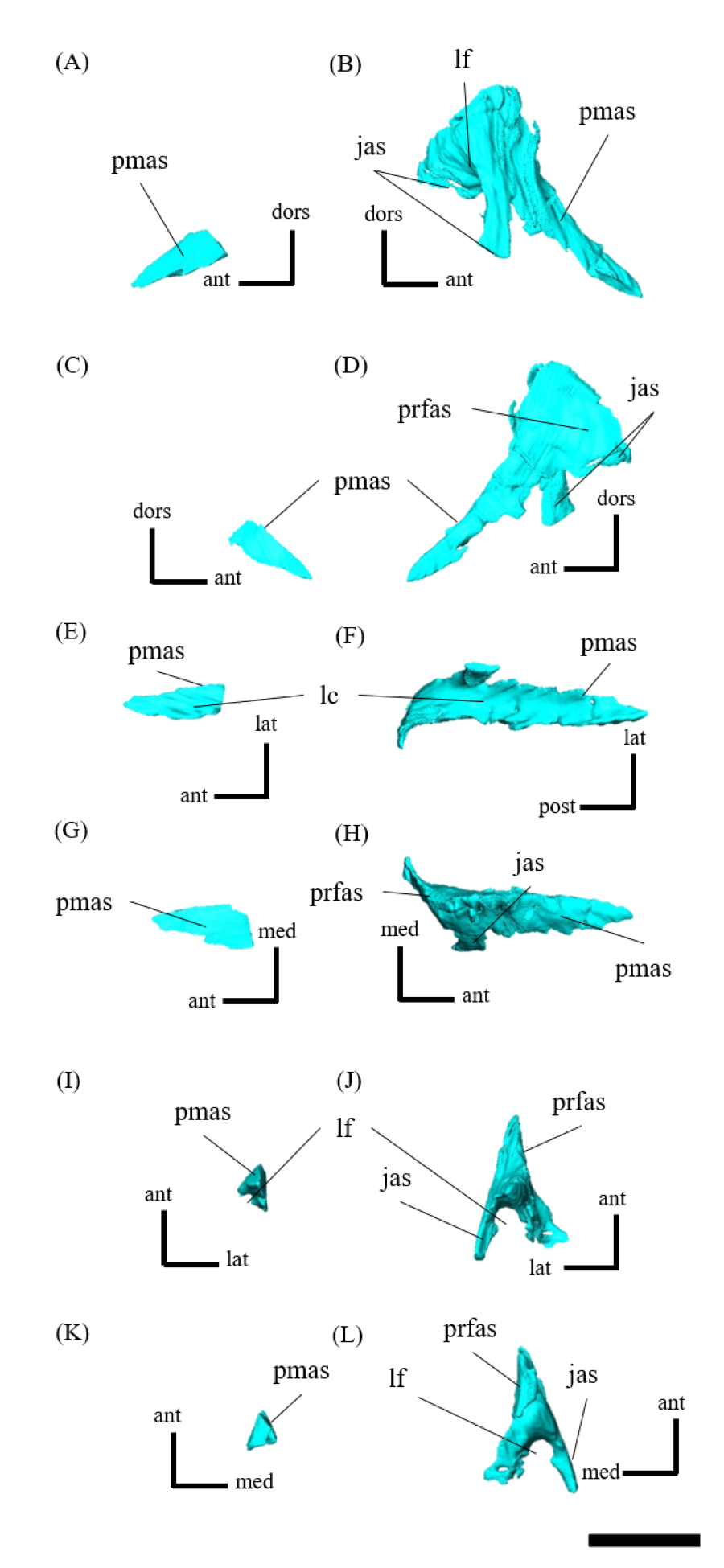

Supplement: Supplemental Information 6 — Abbreviations: jas, jugal articular surface; lc, lacrimal canal; lf, lacrimal foramen; pmas, premaxillary articular surface; prfas, prefrontal articular surface. Scale bar equals 10 mm. [file peerj-13-19547-s006.png]

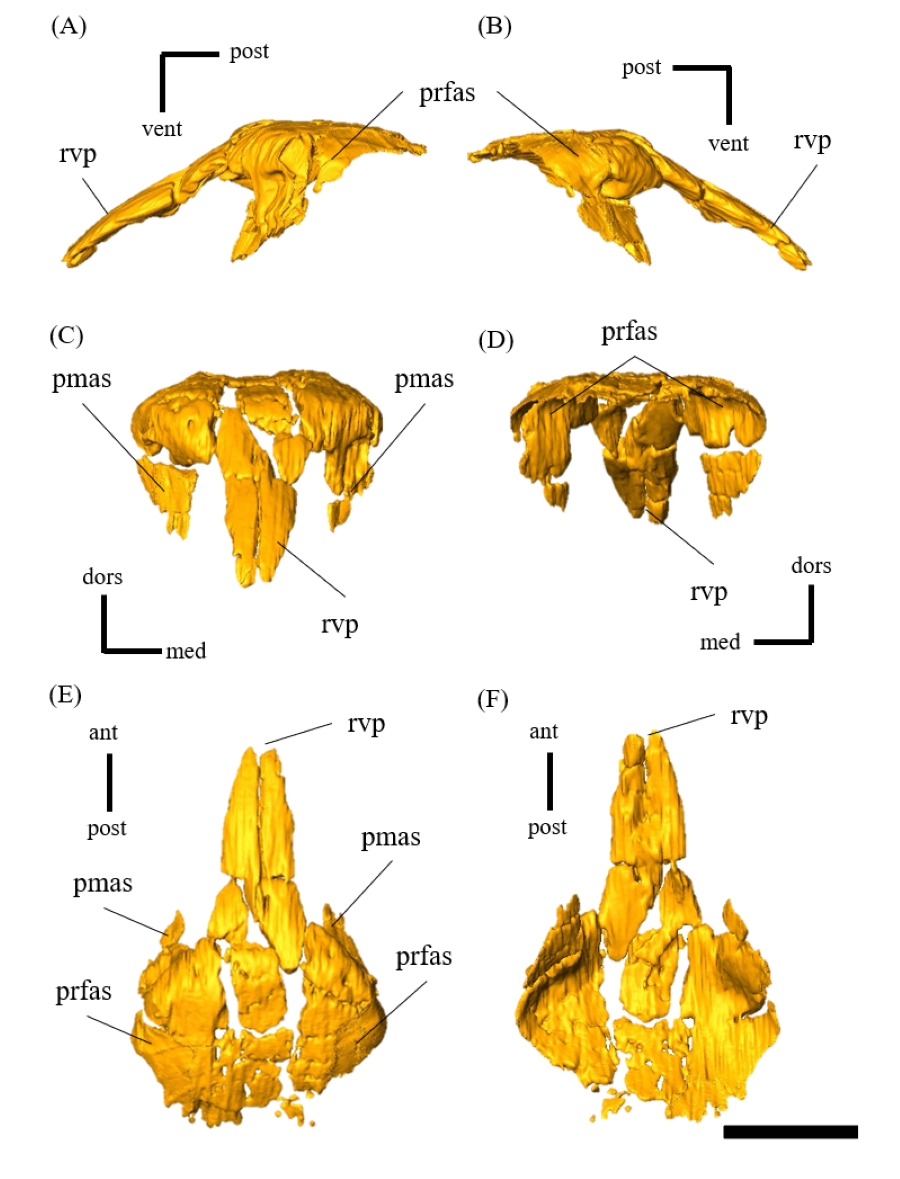

Supplement: Supplemental Information 7 — Abbreviations: pfas, prefrontal articular surface; pmas, premaxillary articular surface; rvp; rostroventral process. Scale bar equals 20 mm. [file peerj-13-19547-s007.png]

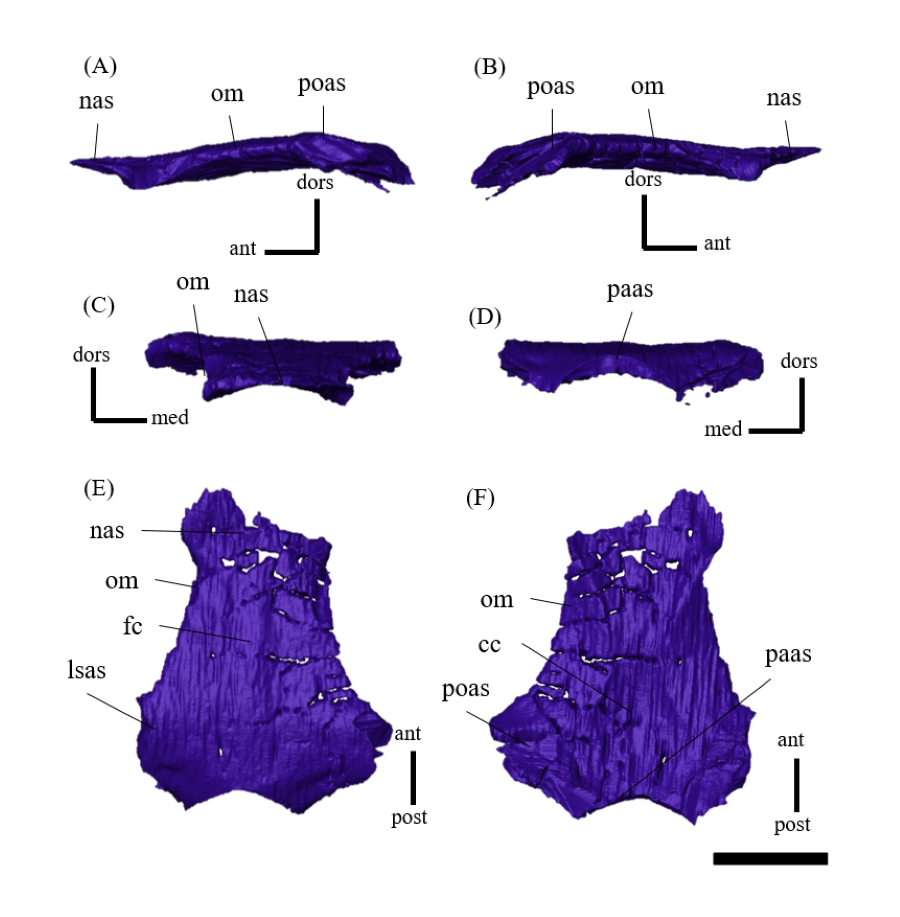

Supplement: Supplemental Information 8 — Abbreviations: cc, cerebral cavity; fc, frontal crest; lsas, laterosphenoid articular surface; nas, nasal articular surface; om, orbital margin; paas, parietal articular surface; poas, postorbital articular surface. Scale bar equals 20 mm. [file peerj-13-19547-s008.png]

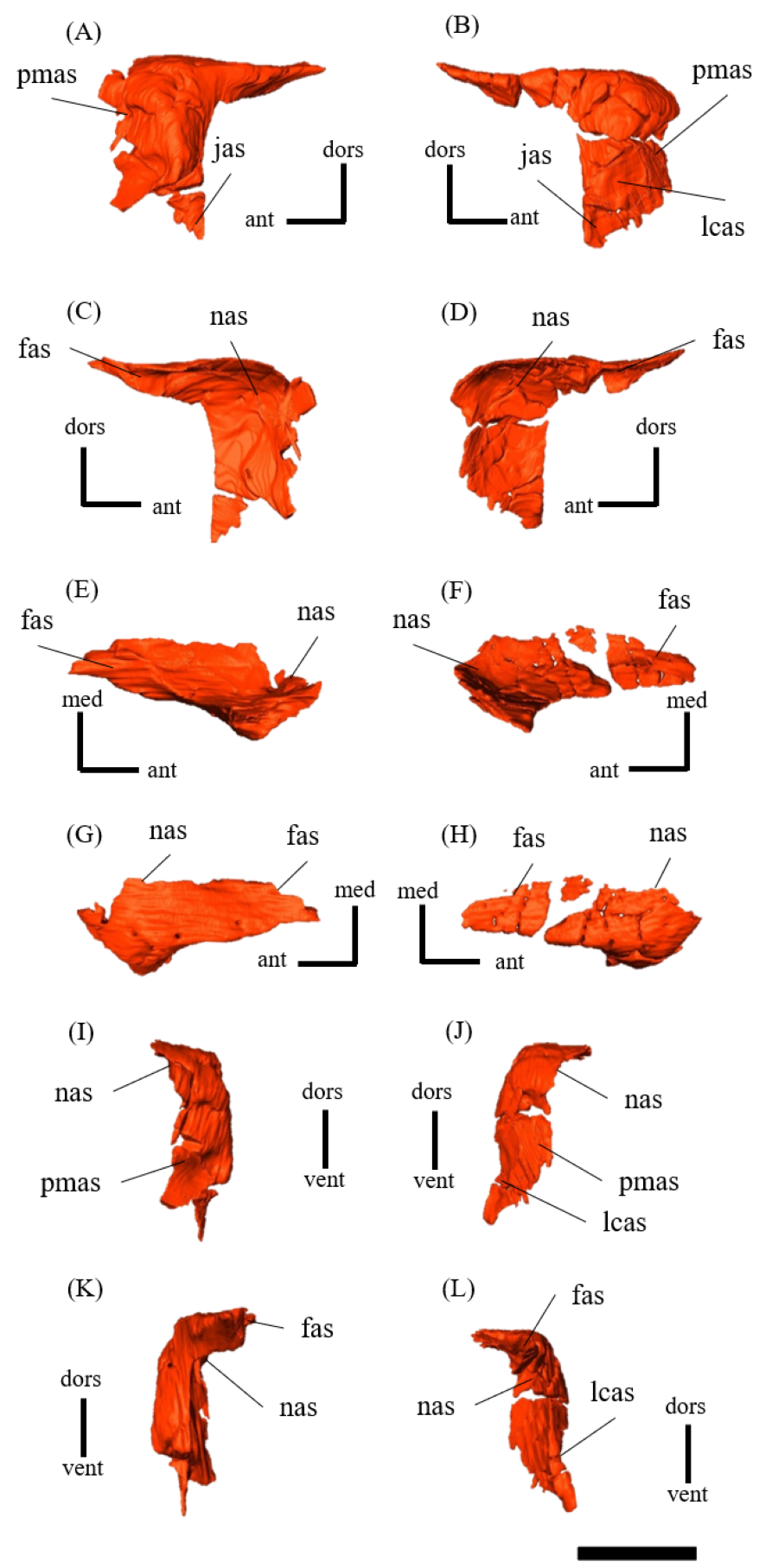

Supplement: Supplemental Information 9 — Abbreviations: fas, frontal articular surface; jas, jugal articular surface; lcas, lacrimal articular surface; nas, nasal articular surface; pmas, premaxillary articular surface. Scale bar equals 20 mm. [file peerj-13-19547-s009.png]

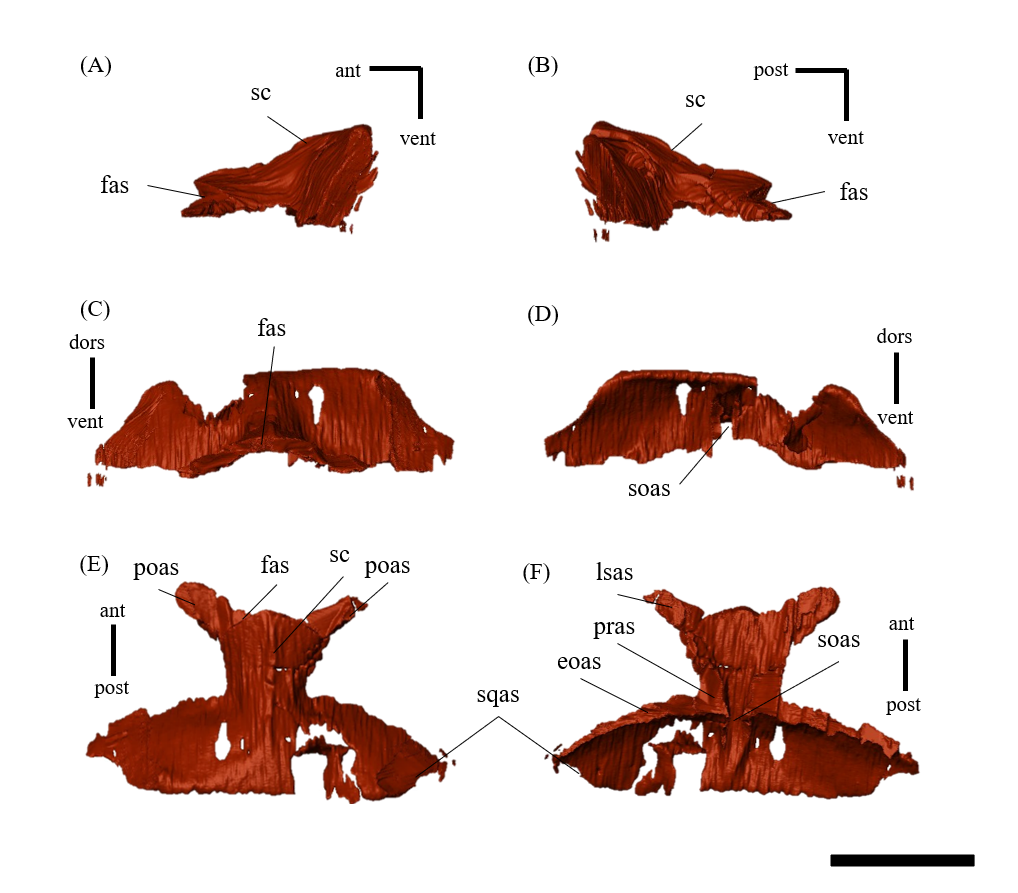

Supplement: Supplemental Information 10 — Abbreviations: eoas, exoccipital articular surface; fas, frontal articular surface; lsas, laterosphenoid articular surface; pras, prootic articular surface; sc, sagittal crest; soas, supraoccipital articular surface; sqas, squamosal articular surface. Scale bar equals 30 mm. [file peerj-13-19547-s010.png]

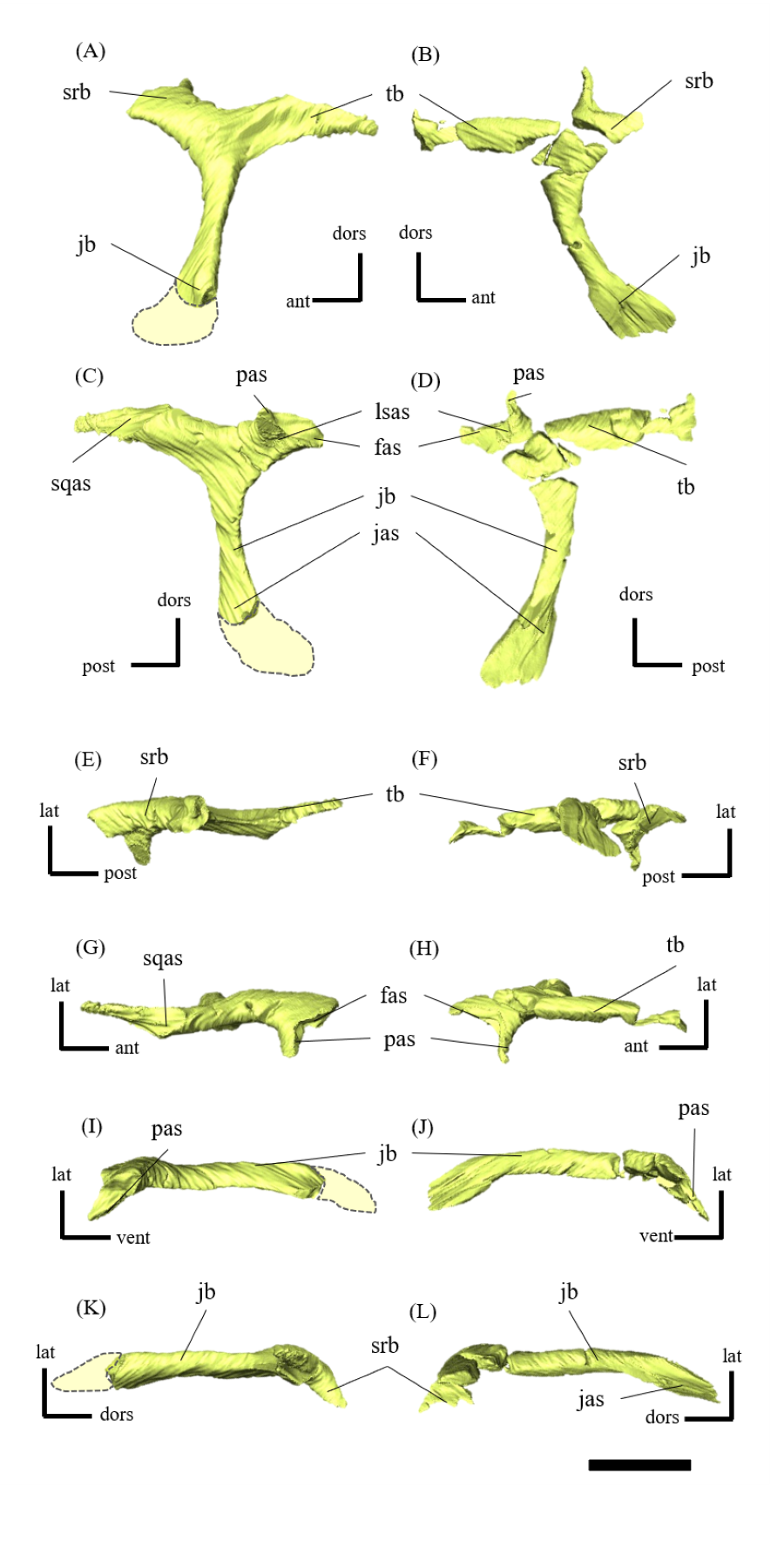

Supplement: Supplemental Information 11 — Black dotted line indicates the missing part. Abbreviations: fas, frontal articular surface; jas, jugal articular surface; jb, jugal bar of postorbital; lsas, laterosphenoid articular surface; pas, parietal articular surface; sqas, squamosal articular surface; srb, skull roof bar of postorbital; tb, temporal bar of postorbital. Scale bar equals 20 mm. [file peerj-13-19547-s011.png]

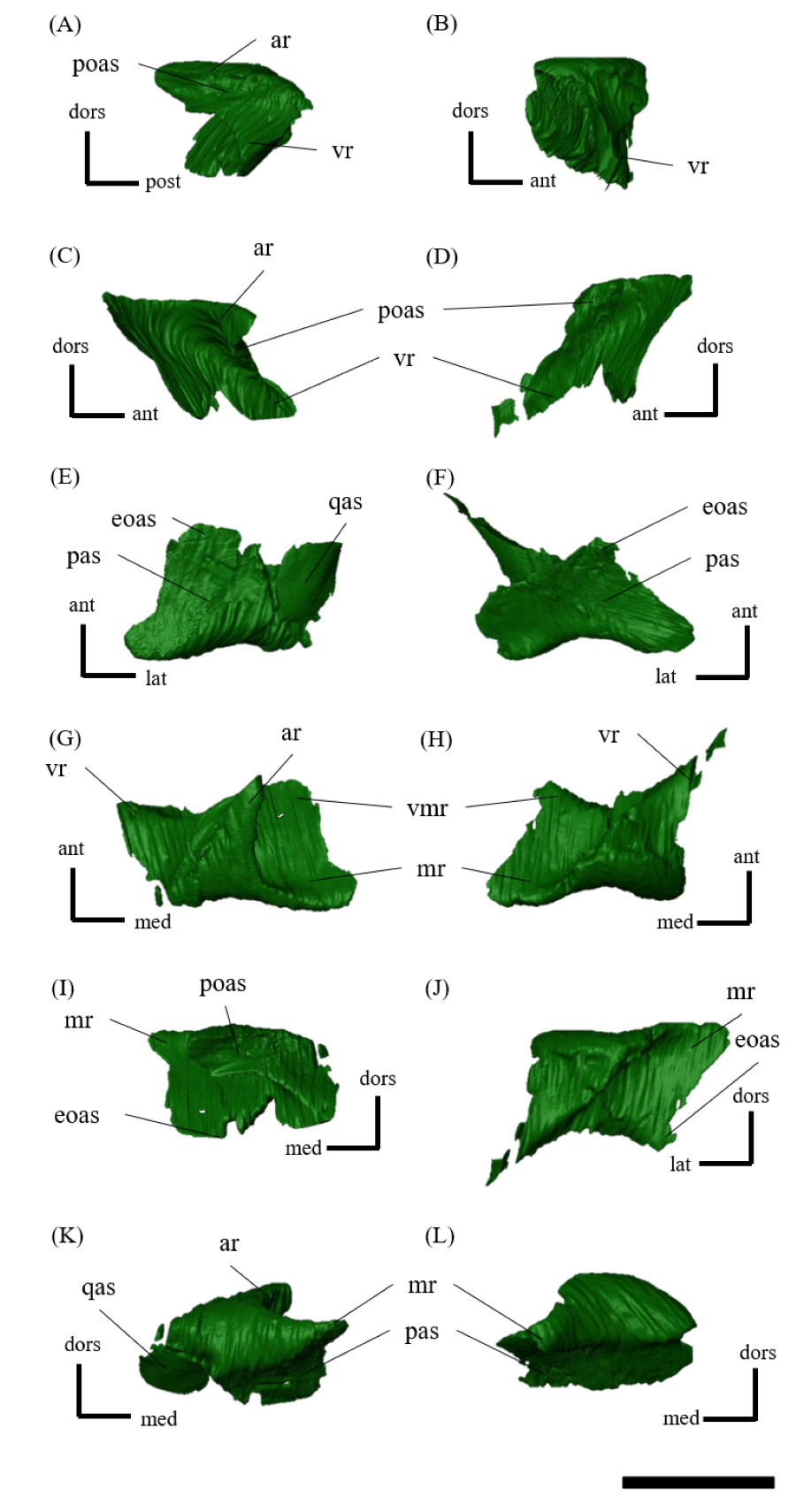

Supplement: Supplemental Information 12 — Abbreviations: ar, anterior ramus; eoas, exoccipital articular surface; mr, medial ramus; pas, parietal articular surface; poas, postorbital articular surface; qas, quadrate surface; vmr, ventromedial ramus; vr, ventral ramus. Scale bar equals 20 mm. [file peerj-13-19547-s012.png]

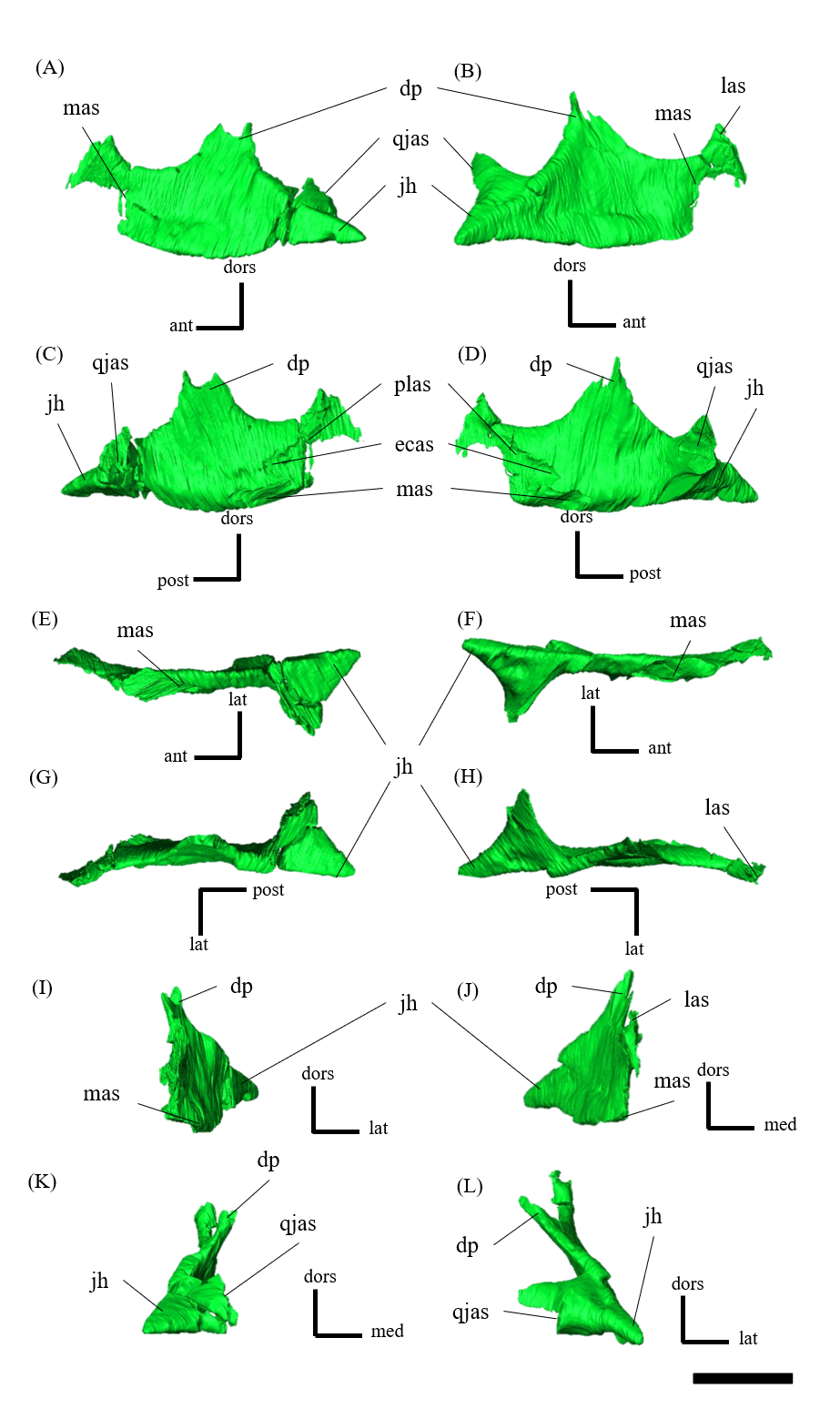

Supplement: Supplemental Information 13 — Abbreviations: dp, dorsal process; ecas, ectopterygoid articular surface; jh, jugal horn; las, lacrimal articular surface; mas, maxillary articular surface; plas, palatine articular surface; qjas, quadratojugal articular surface. Scale bar equals 30 mm. [file peerj-13-19547-s013.png]

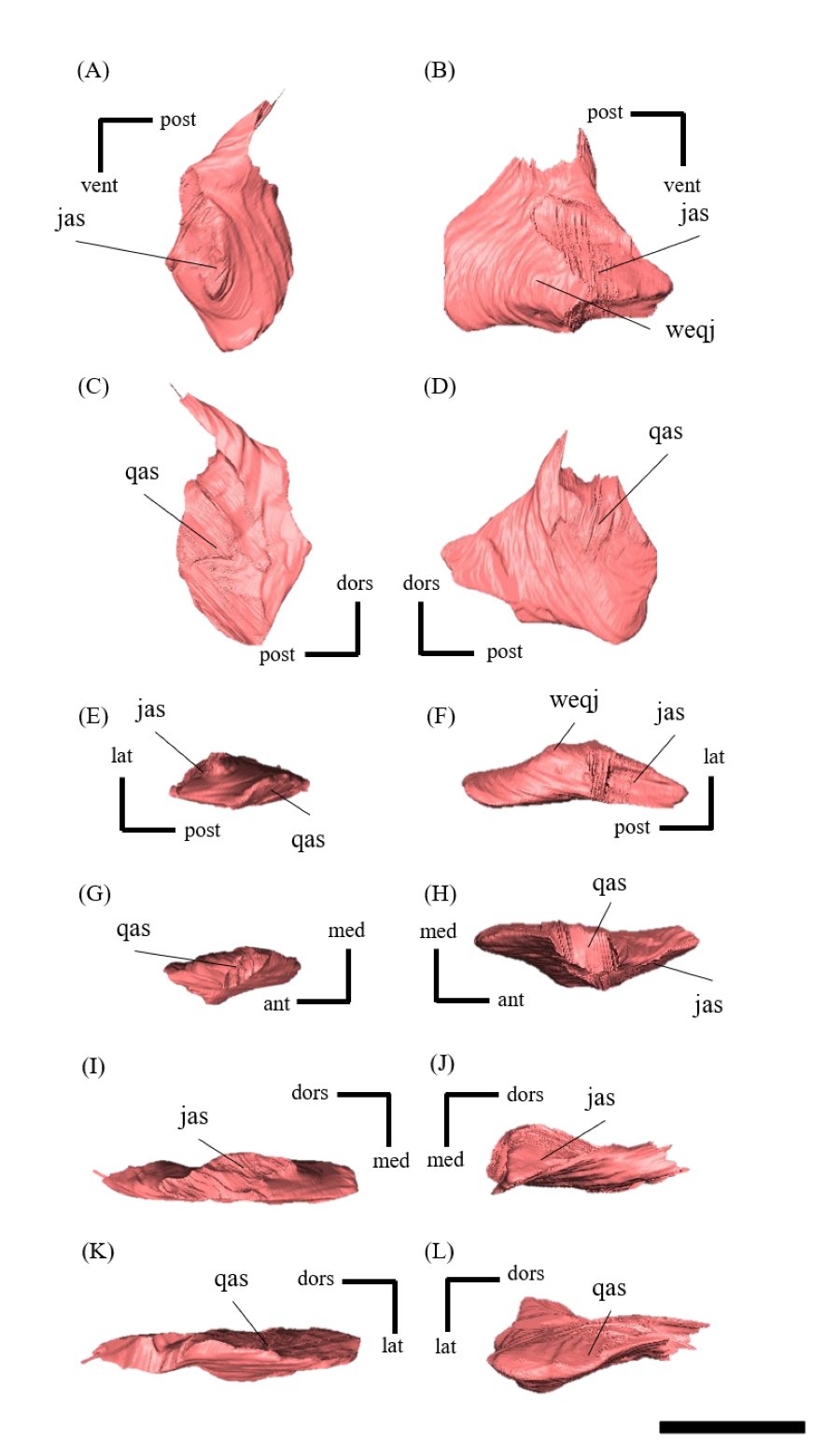

Supplement: Supplemental Information 14 — Abbreviations: jas, jugal articular surface; qas, quadrate articular surface; weqj, weak eminence of quadratojugal. Scale bar equals 20 mm. [file peerj-13-19547-s014.png]

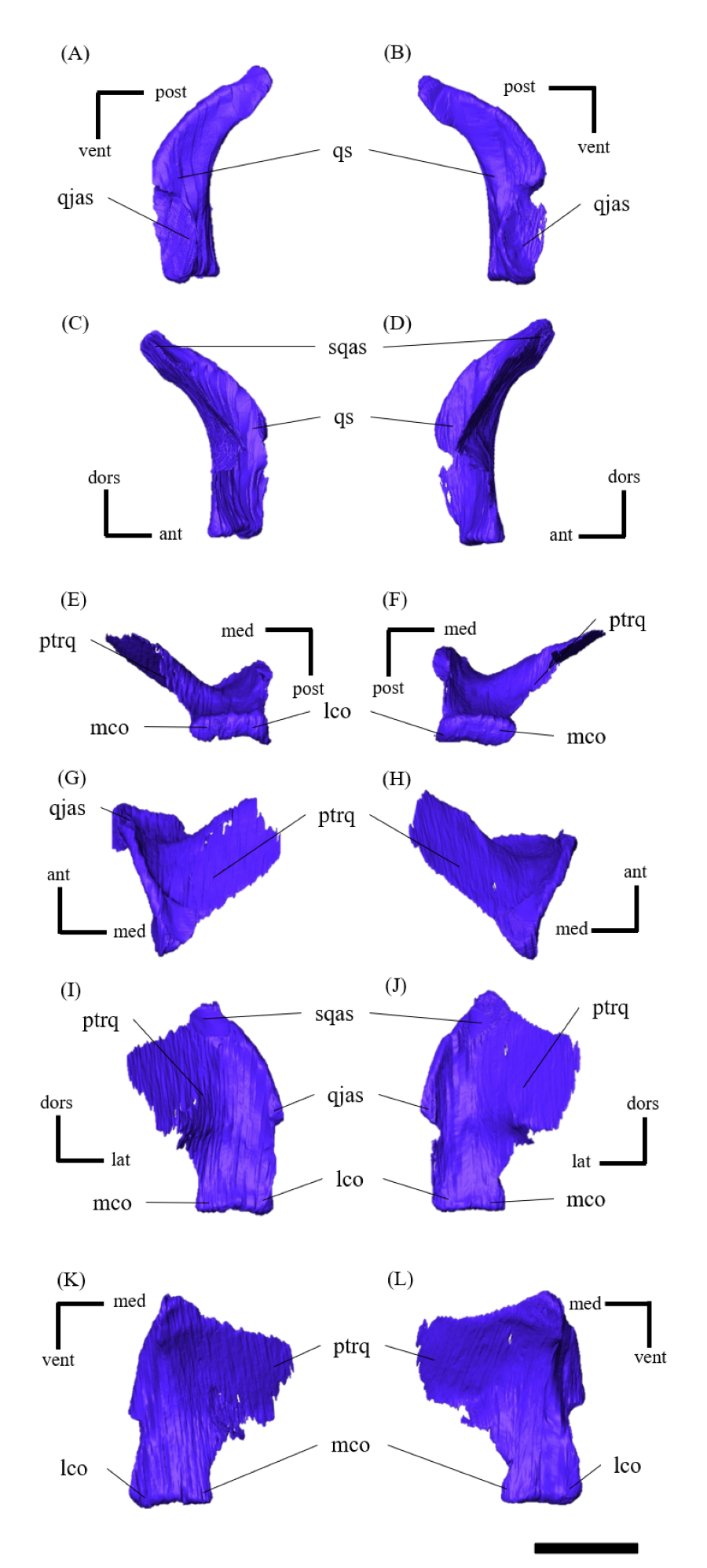

Supplement: Supplemental Information 15 — Abbreviations: lco, lateral condyle; mco, medial condyle; ptrq, pterygoid ramus of quadrate; qjas, quadratojugal articular surface; qs, quadrate shaft; sqas, squamosal articular surface. Scale bar equals 20 mm. [file peerj-13-19547-s015.png]

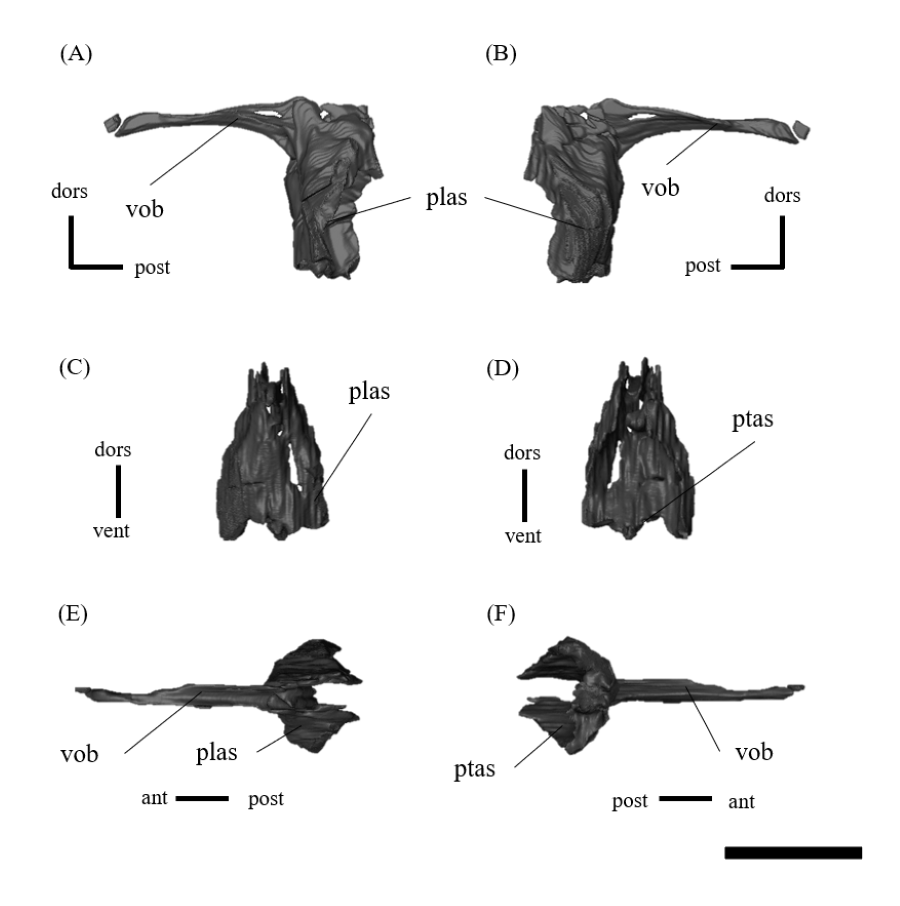

Supplement: Supplemental Information 16 — Abbreviations: plas, palatine articular surface; ptas, pterygoid articular surface; vob, vomerine bar. Scale bar equals 20 mm. [file peerj-13-19547-s016.png]

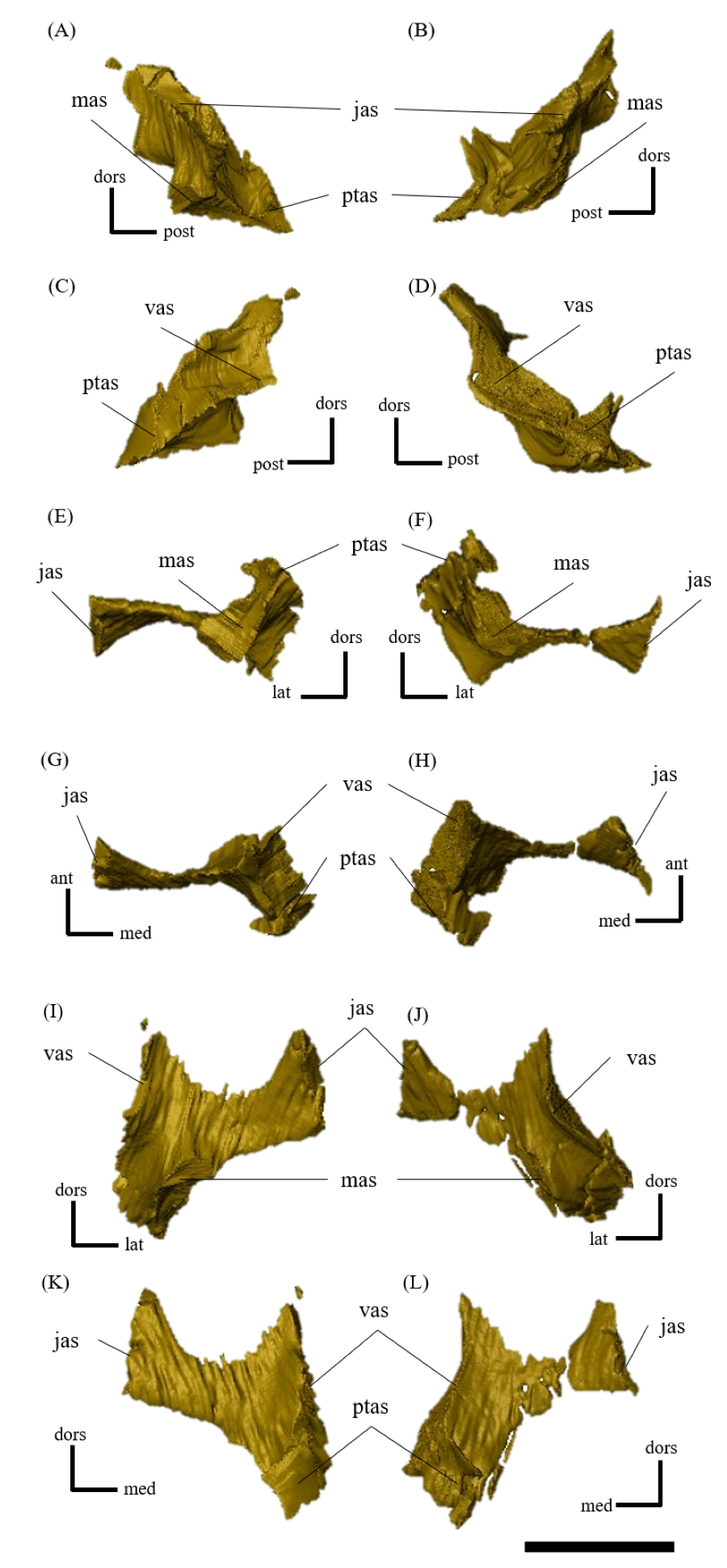

Supplement: Supplemental Information 17 — Abbreviations: jas, jugal articular surface; mas, maxillary articular surface; ptas, pterygoid articular surface; vas, vomeral articular surface. Scale bar equals 20 mm. [file peerj-13-19547-s017.png]

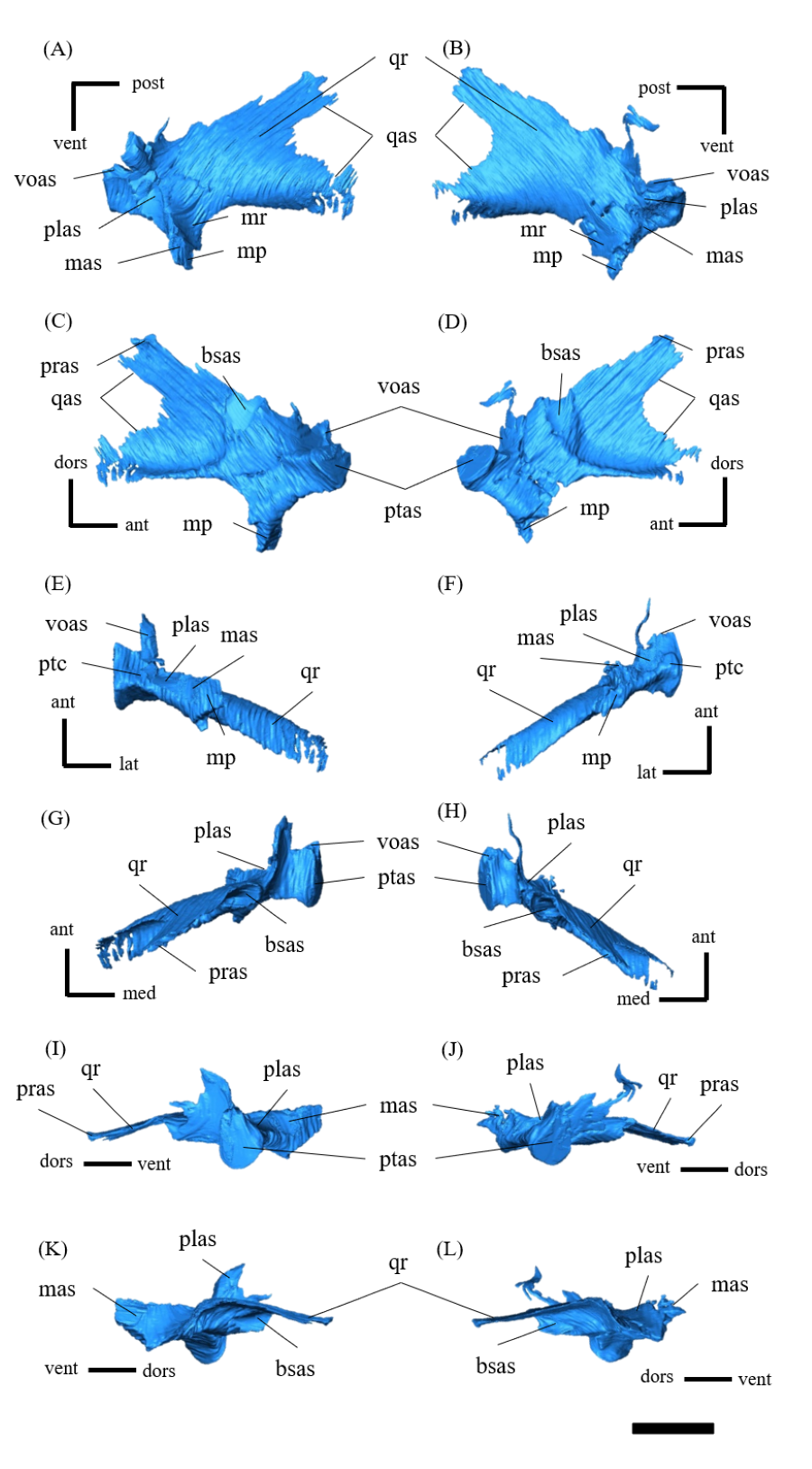

Supplement: Supplemental Information 18 — Abbreviations: bsas, basisphenoid articular surface; mas, maxillary articular surface; mp, mandibular process; mr; mandibular ramus; plas, palatine surface area; pras, prootic articular surface; ptas, pterygoid articular surface; qas, quadrate articular surface; qr, quadrate ramus of pterygoid, voas, vomeral articular surface. Scale bar equals 20 mm. [file peerj-13-19547-s018.png]

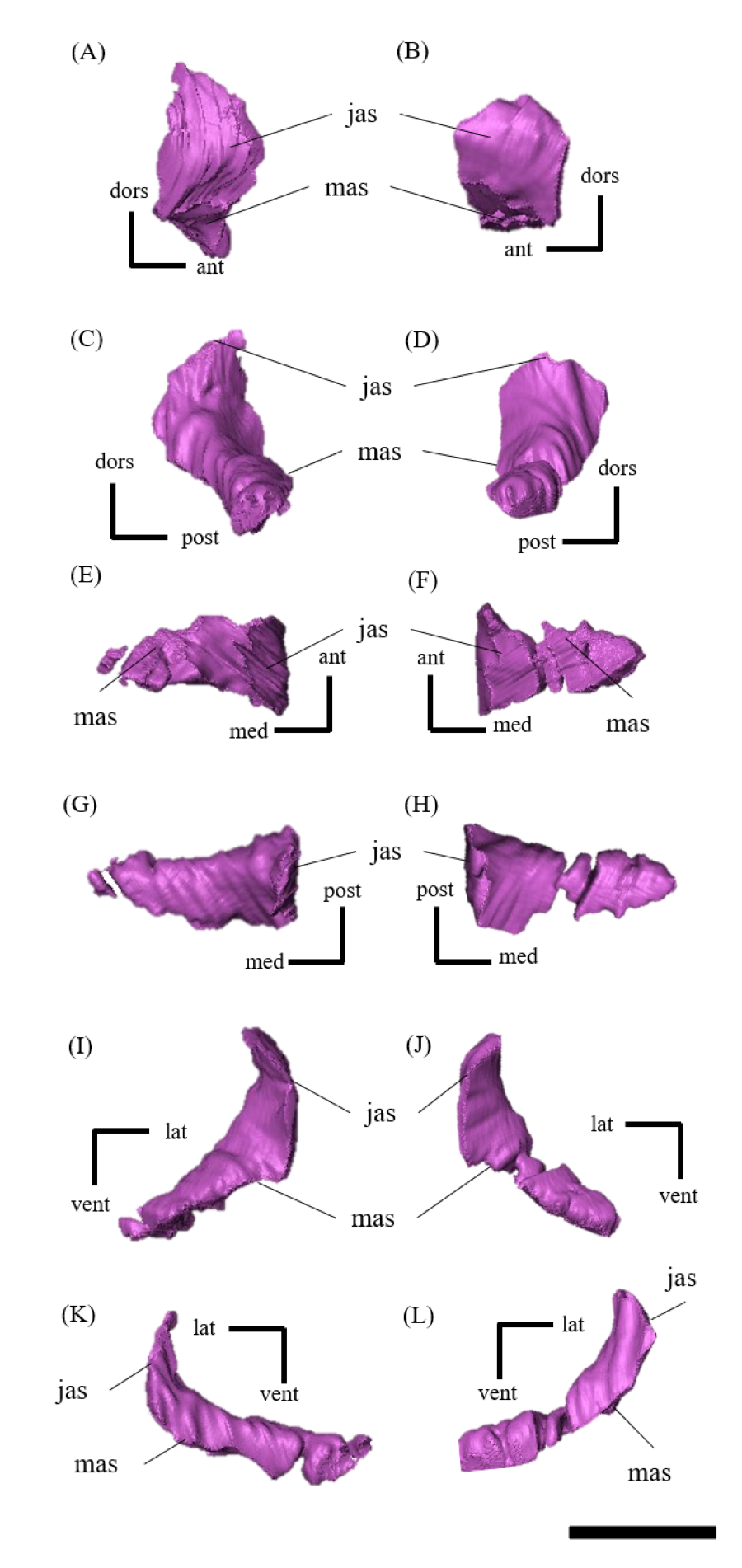

Supplement: Supplemental Information 19 — Abbreviations: jas, jugal articular surface; mas, maxillary articular surface. Scale bar equals 10 mm. [file peerj-13-19547-s019.png]

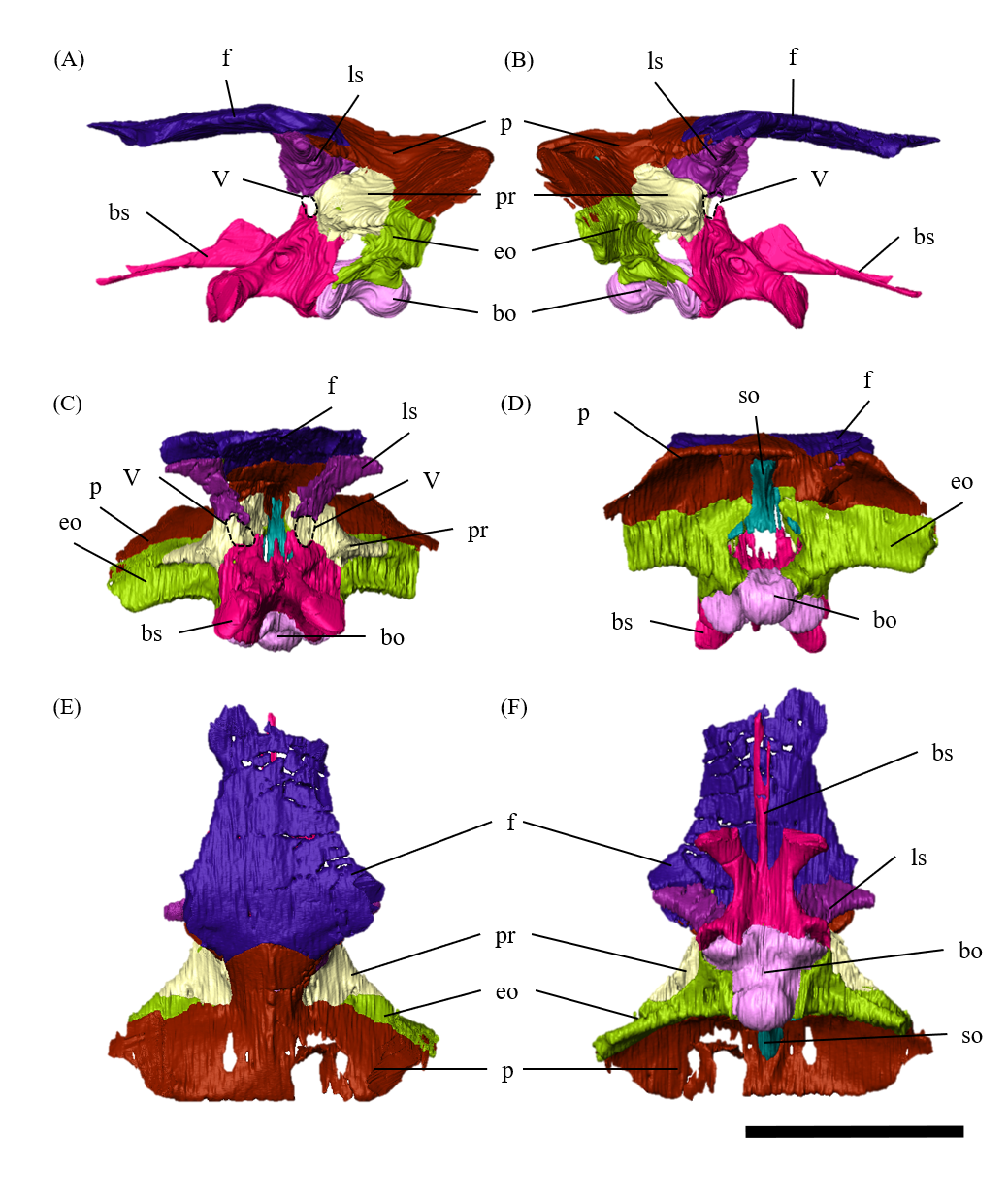

Supplement: Supplemental Information 20 — Abbreviations: bo, basioccipital; bs, basisphenoid; eo, exoccipital; f, frontal; ls, laterosphenoid; p, parietal; so, supraoccipital; V, trigeminal foramen. Scale bar equals 50 mm. [file peerj-13-19547-s020.png]

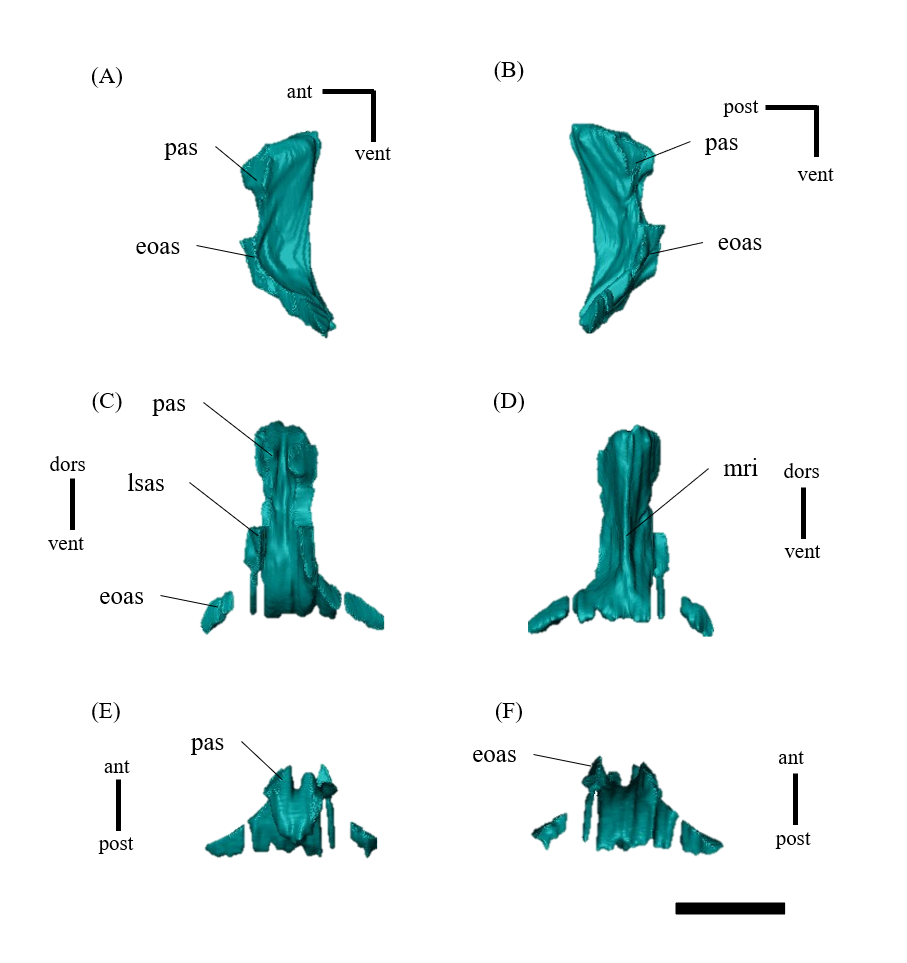

Supplement: Supplemental Information 21 — Abbreviations: eoas, exoccipital articular surface; lsas, laterosphenoid articular surface; mri, medial ridge; pas, parietal articular surface. Scale bar equals 10 mm. [file peerj-13-19547-s021.png]

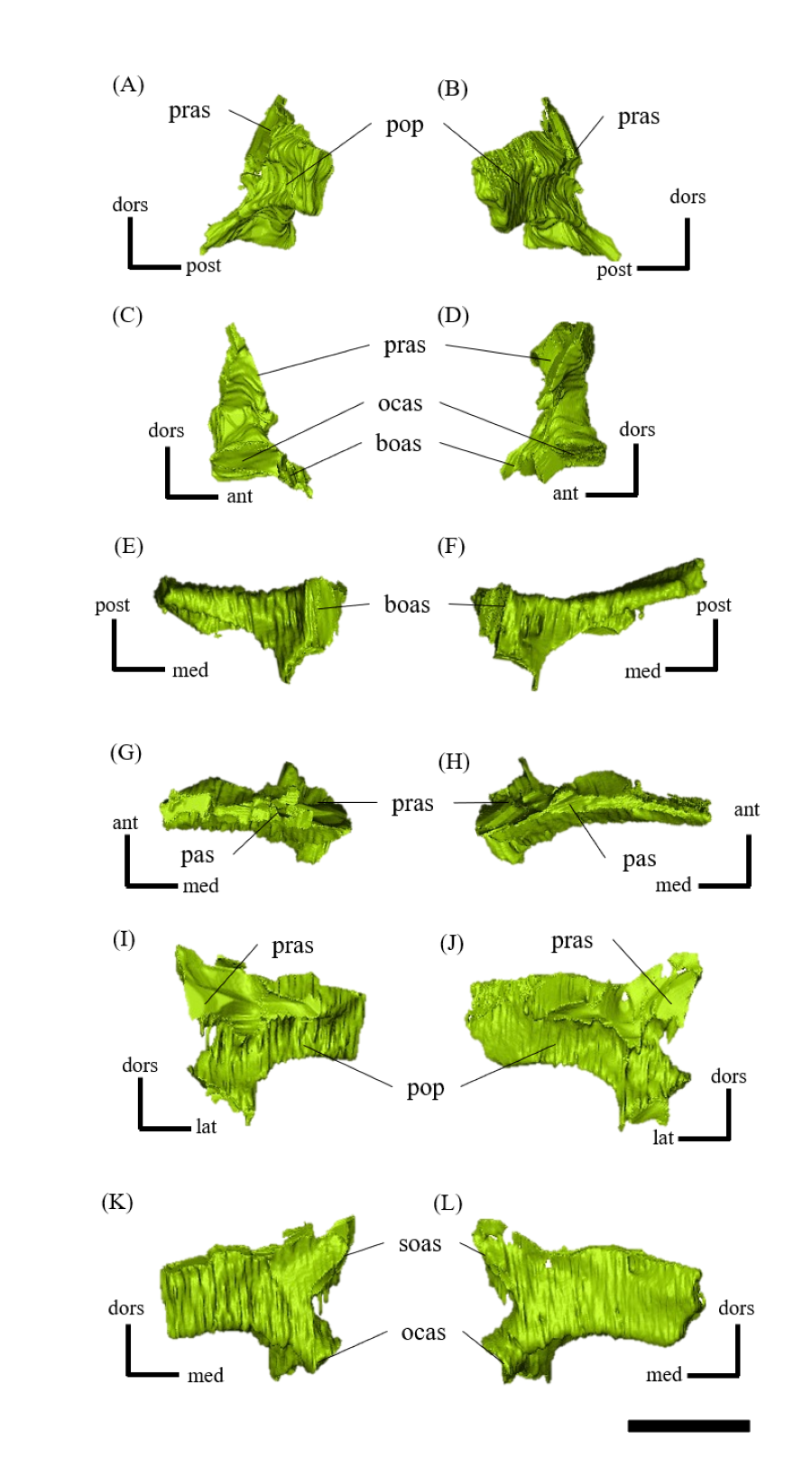

Supplement: Supplemental Information 22 — Abbreviations: boas, basioccipital articular surface; ocas, occipital condyle articular surface; pas, parietal articular surface; pop, paroccipital process; pras, prootic articular surface; soas, supraoccipital articular surface. Scale bar equals 20 mm. [file peerj-13-19547-s022.png]

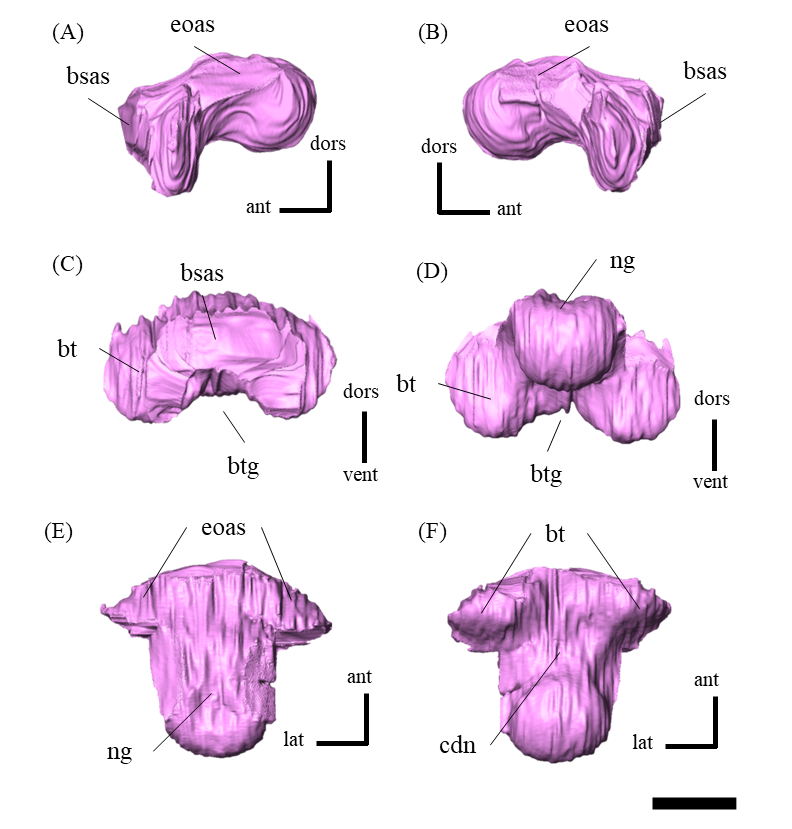

Supplement: Supplemental Information 23 — Abbreviations: bsas, basisphenoid articular surface; bt, basal tubera; btg, basal tubera groove; cdn, condylar neck; eoas, exoccipital articular surface; ng, neural groove. Scale bar equals 10 mm. [file peerj-13-19547-s023.png]

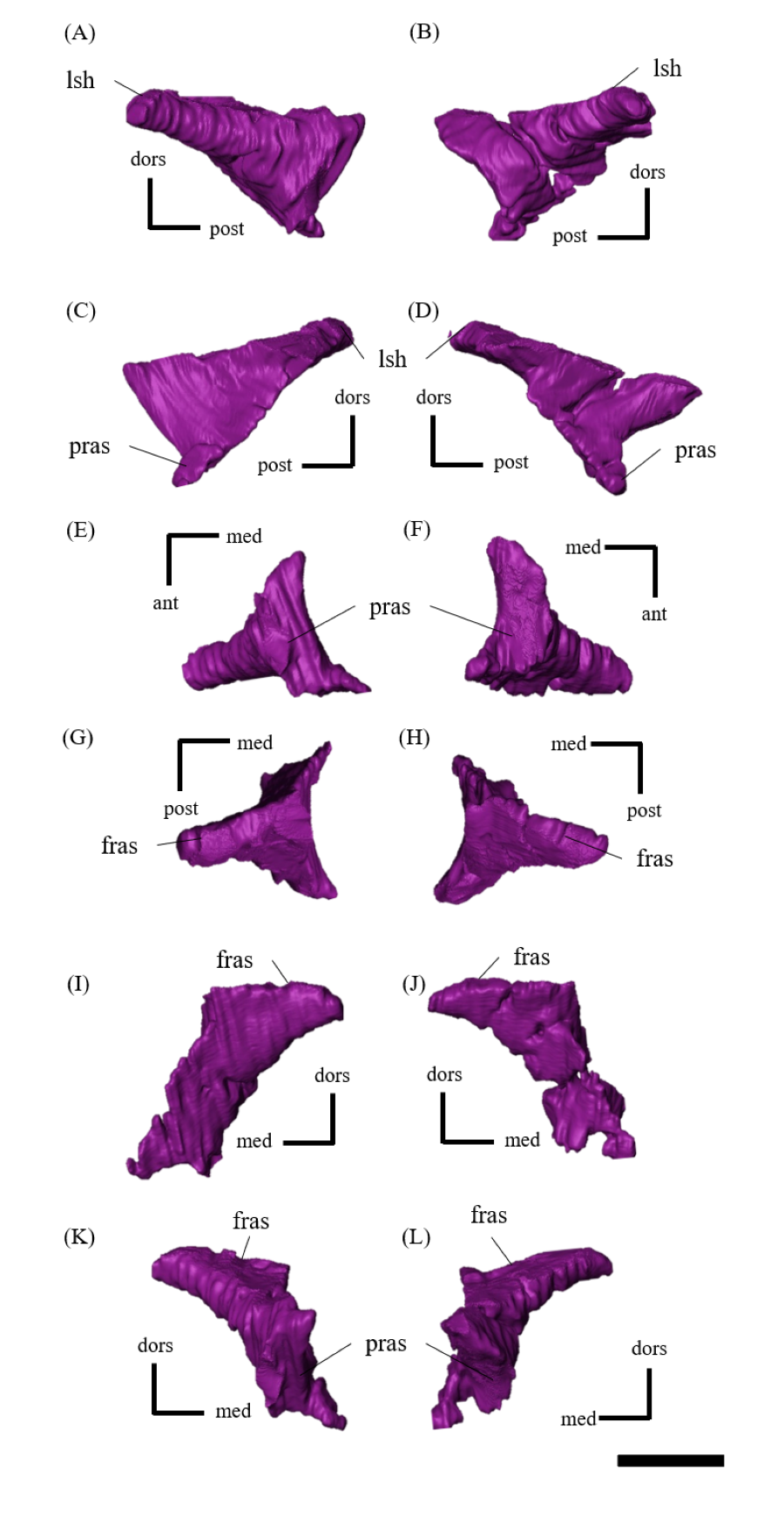

Supplement: Supplemental Information 24 — Abbreviations: fras, frontal articular surface; lsh, laterosphenoid head; pras, prootic articular surface. Scale bar equals 10 mm. [file peerj-13-19547-s024.png]

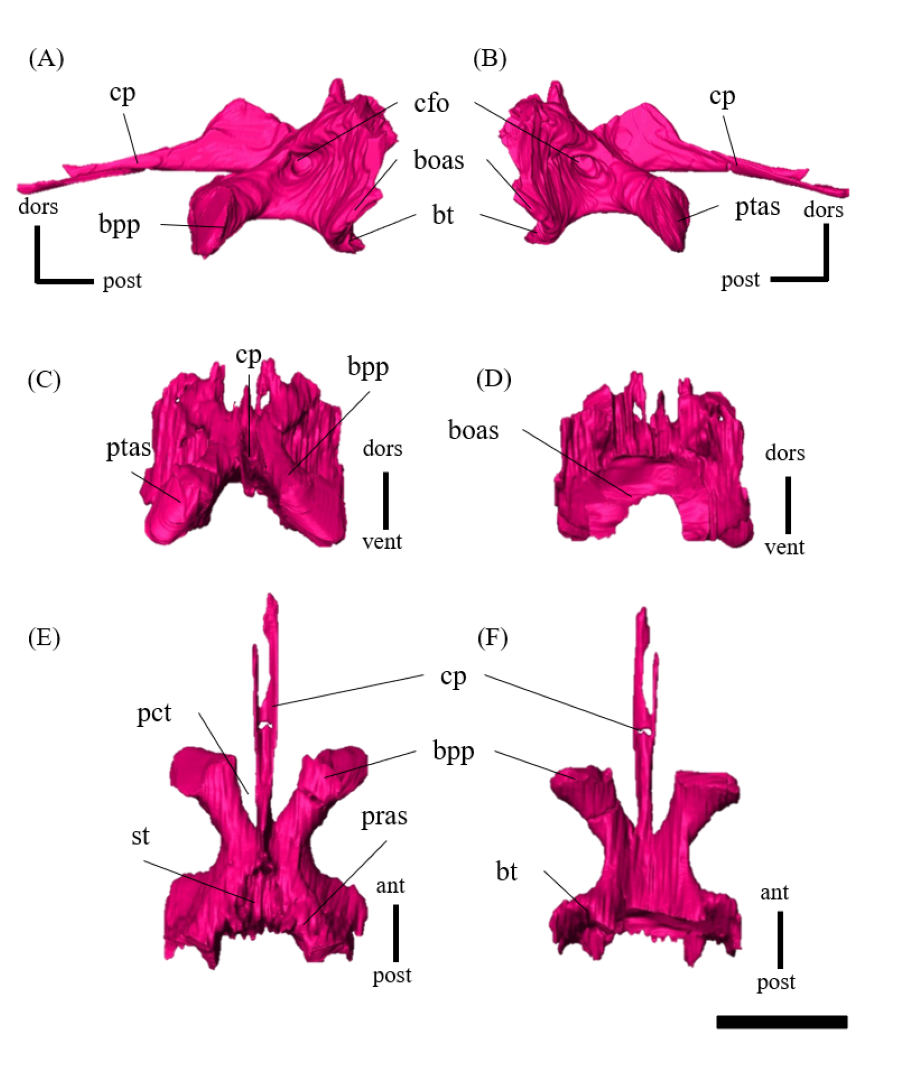

Supplement: Supplemental Information 25 — Abbreviations: boas, basioccipital articular surface; bpp, basipterygoid process; bt, basal tubera; cfo, carotid foramen; cp, cultriform process; pct, paracultriform trough; pras, prootic articular surface; ptas, pterygoid articular surface; st, sella turcica. Scale bar equals 20 mm. [file peerj-13-19547-s025.png]

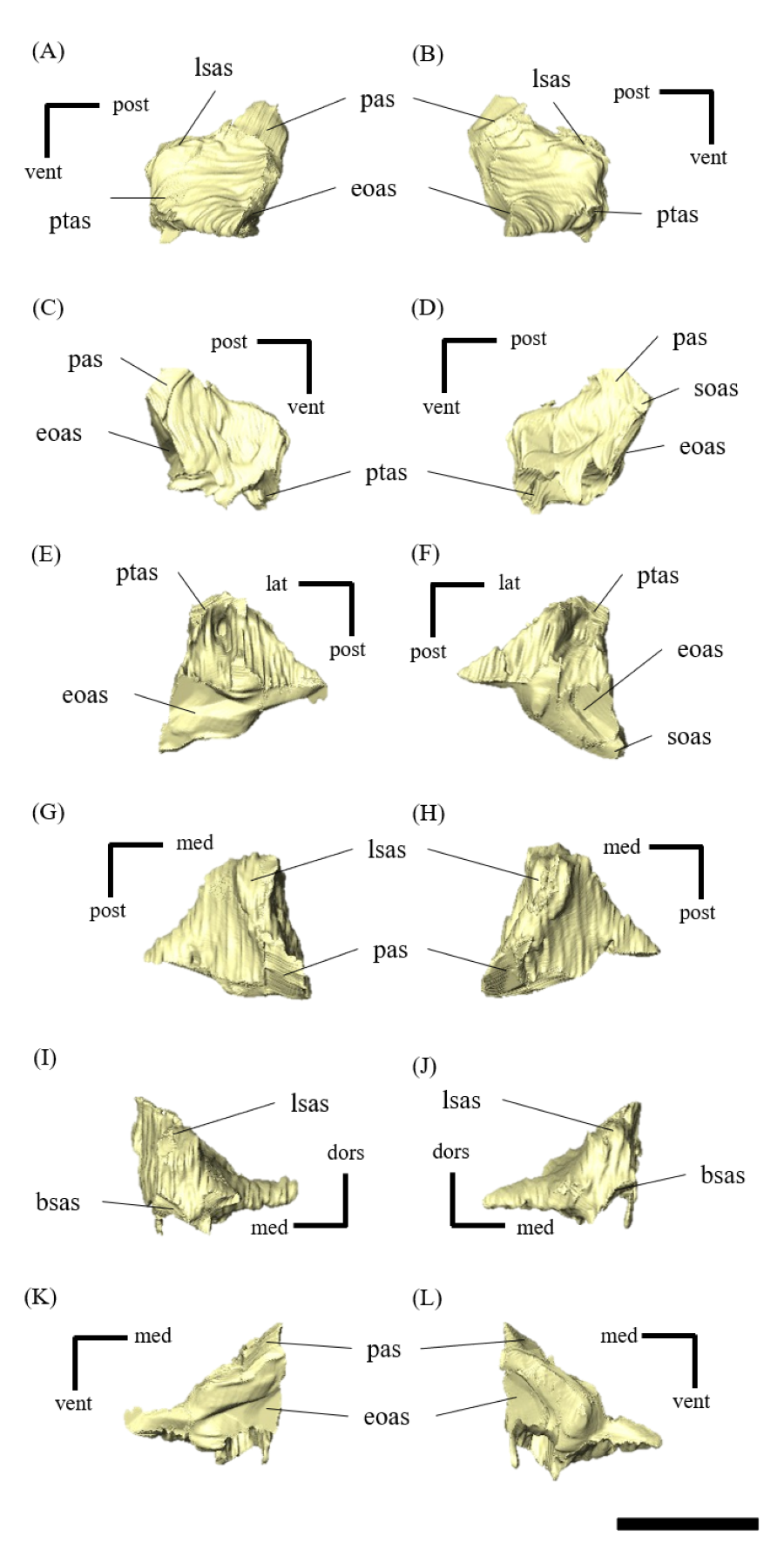

Supplement: Supplemental Information 26 — Abbreviations: bsas, basisphenoid articular surface; eoas, exoccipital articular surface; lsas, laterosphenoid articular surface; pas, parietal articular surface; ptas, pterygoid articular surface; soas, supraoccipital articular surface. Scale bar equals 20 mm. [file peerj-13-19547-s026.png]

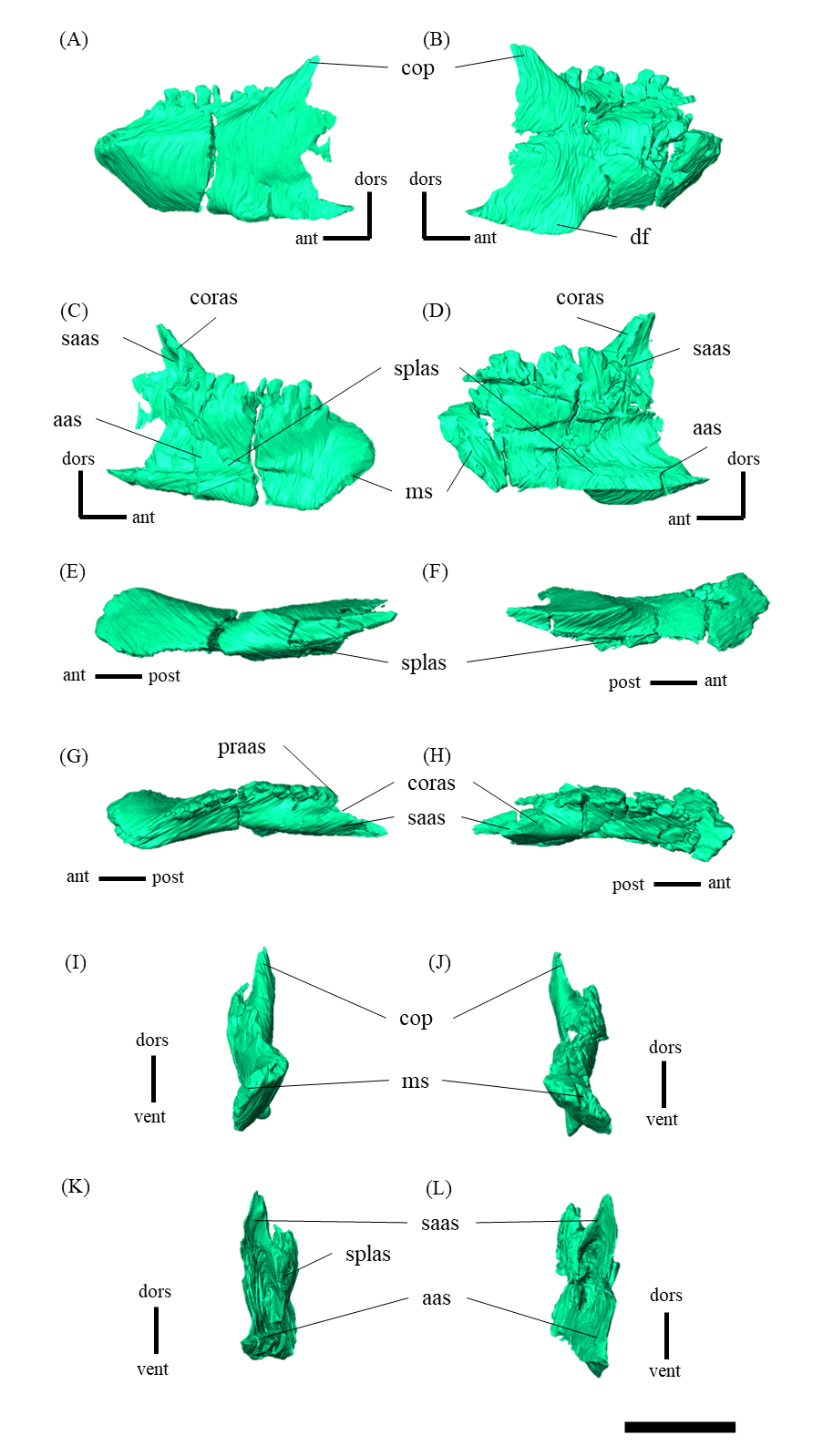

Supplement: Supplemental Information 27 — Abbreviations: aas, angular articular surface; cop, coronoid process; coras, coronoid articular surface; df, dentary flange; ms, maxillary symphysis; praas, prearticular articular surface; saas, surangular articular surface; splas, splenial articular surface. Scale bar equals 30 mm. [file peerj-13-19547-s027.png]

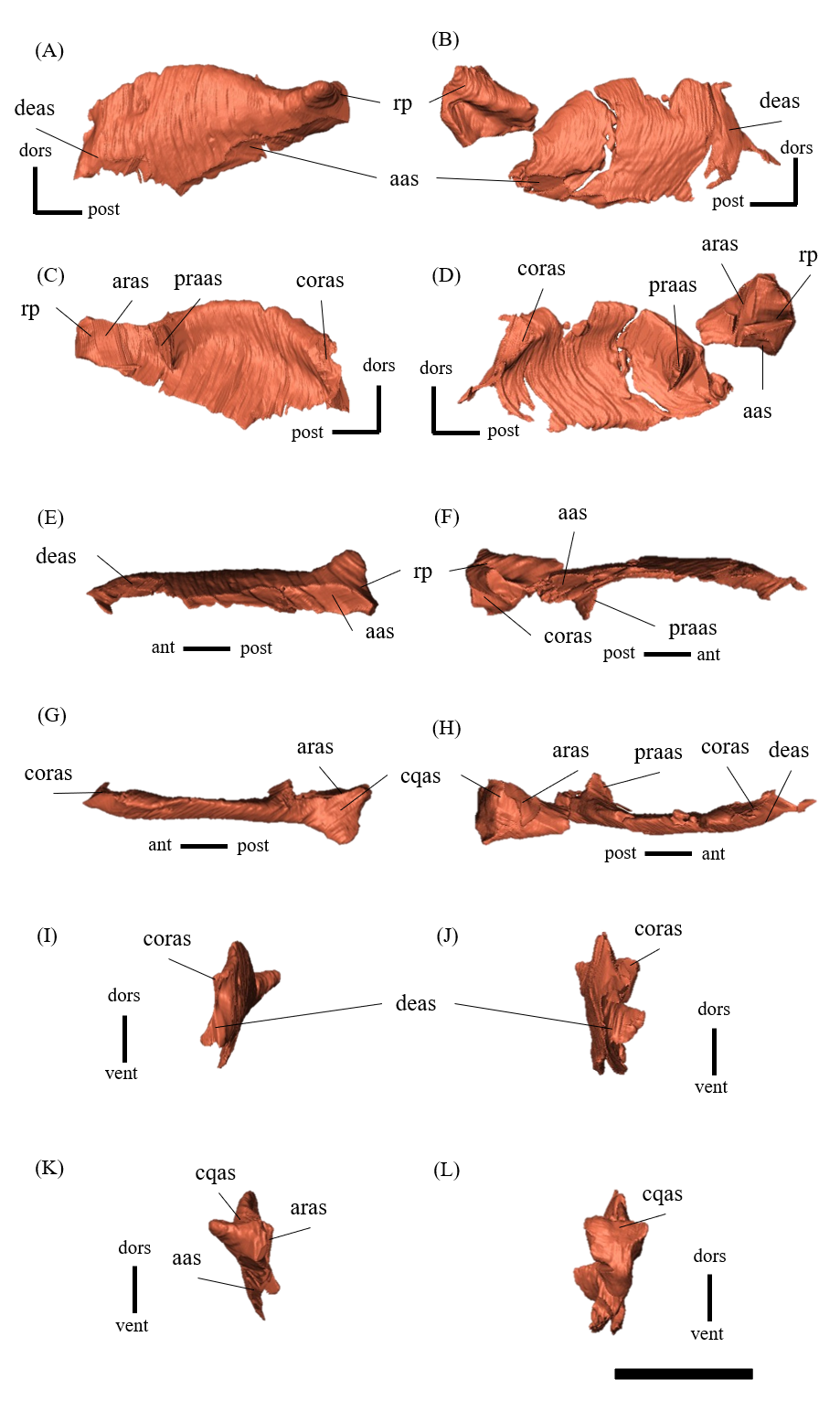

Supplement: Supplemental Information 28 — Abbreviations: aas, angular articular surface; aras, articular articular surface; coras, coronoid articular surface; cqas, articular surface for quadrate condyles; deas, dentary articular surface; praas, prearticular articular surface; rp, retroarticular process. Scale bar equals 30 mm. [file peerj-13-19547-s028.png]

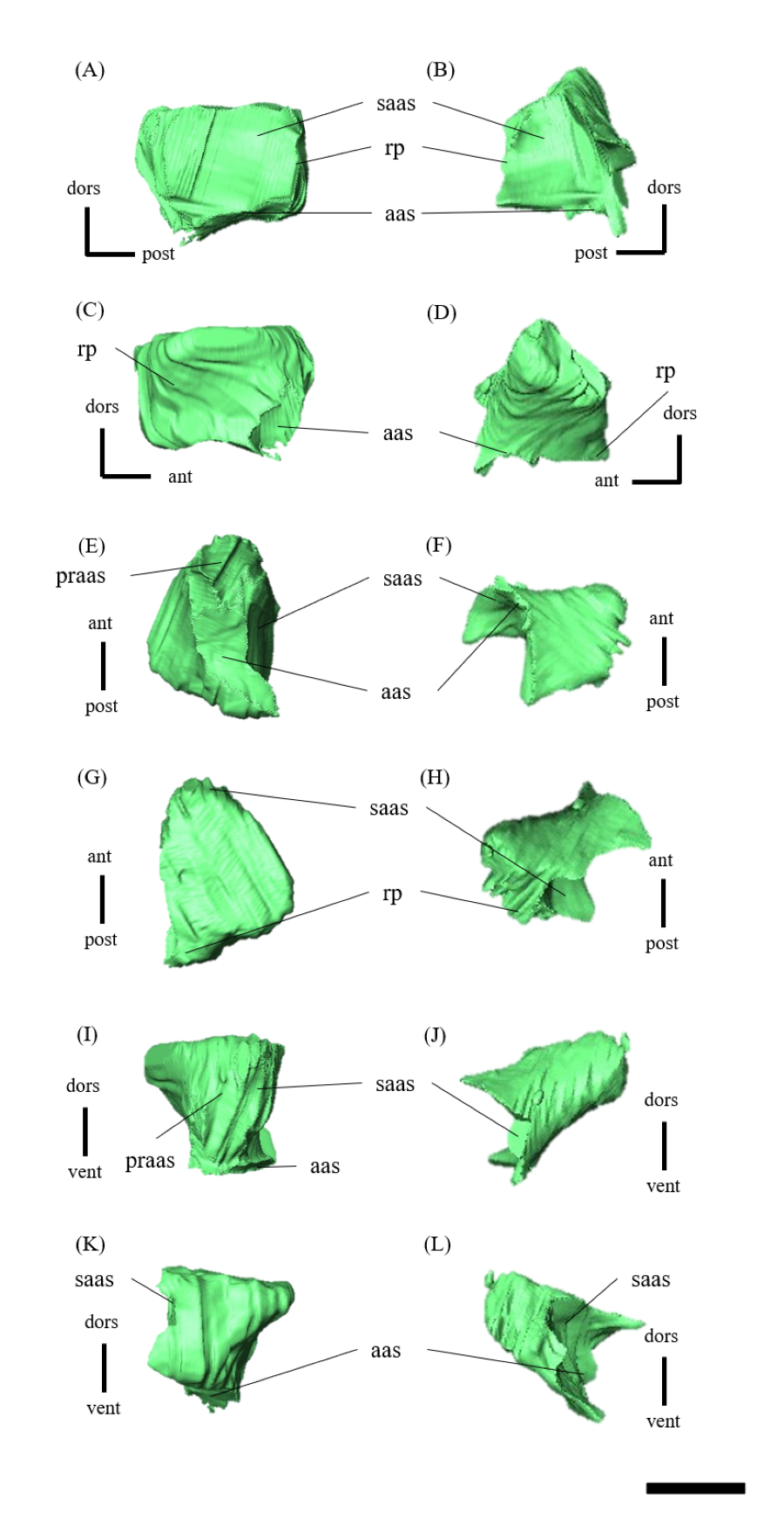

Supplement: Supplemental Information 29 — Abbreviations: aas, articular articular surface; rp, retroarticular process; praas, prearticular articular surface; saas, surangular articular surface. Scale bar equals 10 mm. [file peerj-13-19547-s029.png]

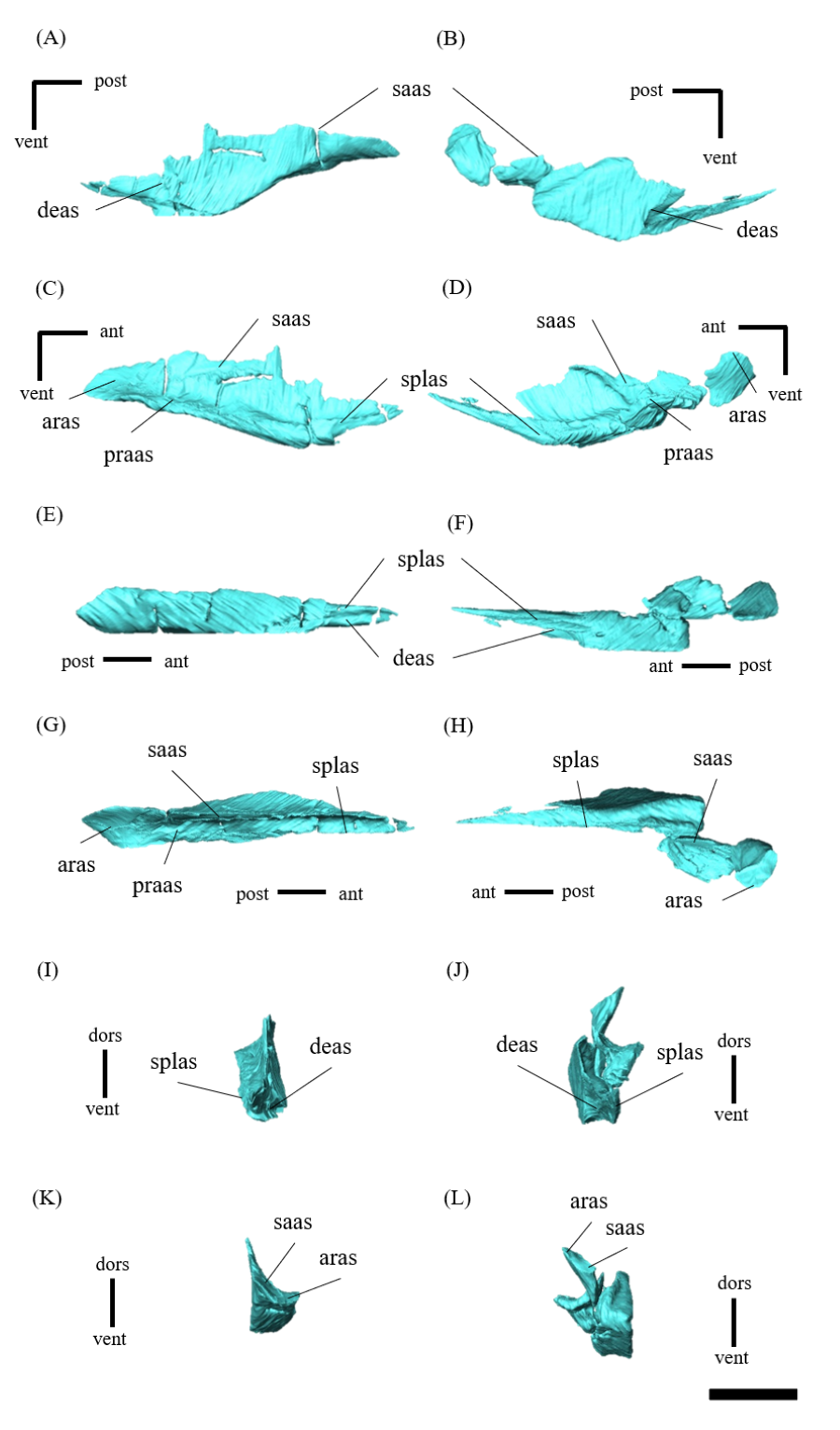

Supplement: Supplemental Information 30 — Abbreviations: aras, articular articular surface; deas, dentary articular surface; praas, prearticular articular surface; saas, surangular articular surface; splas, splenial articular surface. Scale bar equals 20 mm. [file peerj-13-19547-s030.png]

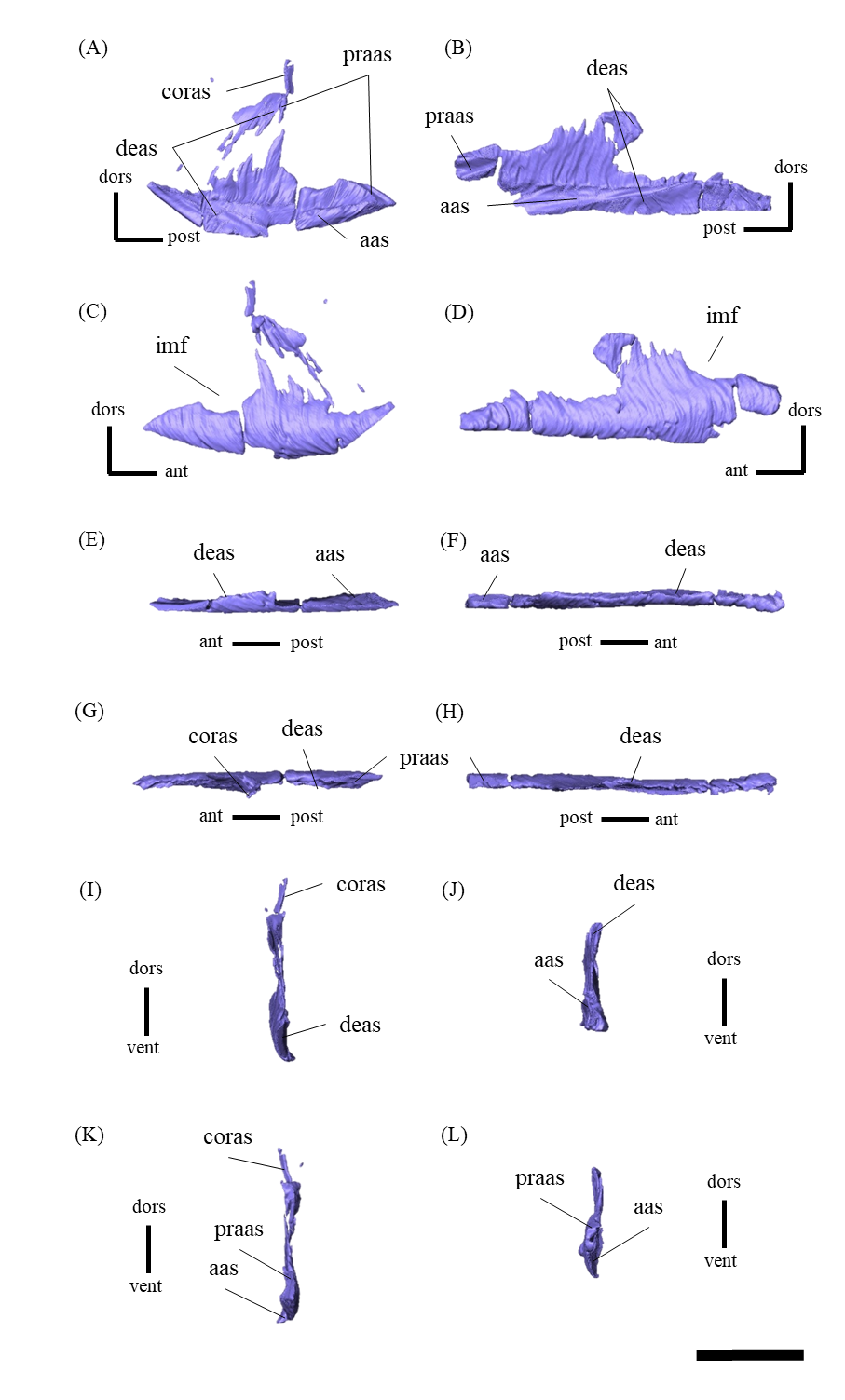

Supplement: Supplemental Information 31 — Abbreviations: aas, angular articular surface; coras, coronoid articular surface; deas, dentary articular surface; imf, internal mandibular fenestra; praas, prearticular articular surface; saas, surangular articular surface. Scale bar equals 20 mm. [file peerj-13-19547-s031.png]

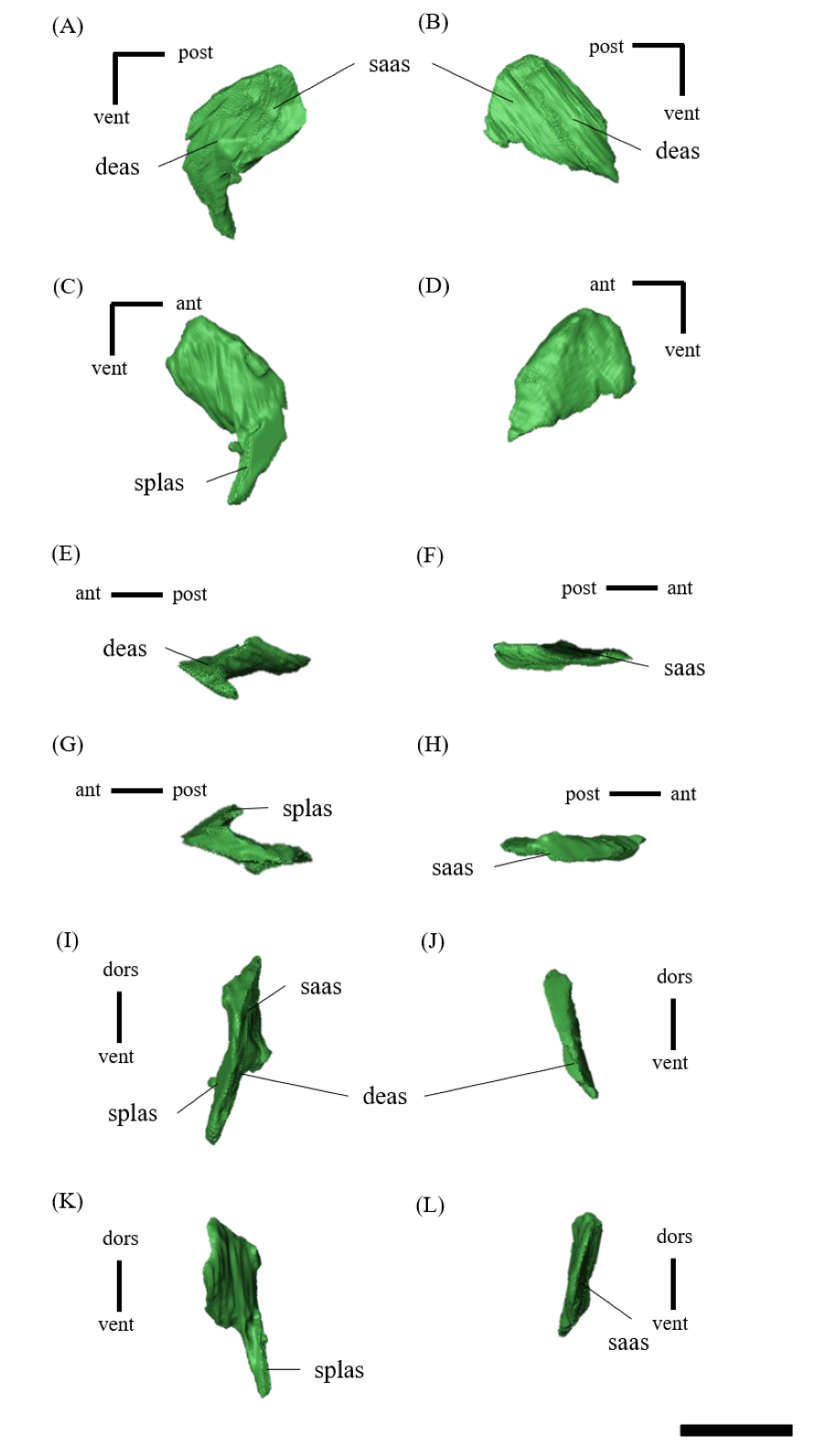

Supplement: Supplemental Information 32 — Abbreviations: deas, dentary articular surface; saas , surangular articular surface; splas, splenial articular surface. Scale bar equals 10 mm. [file peerj-13-19547-s032.png]

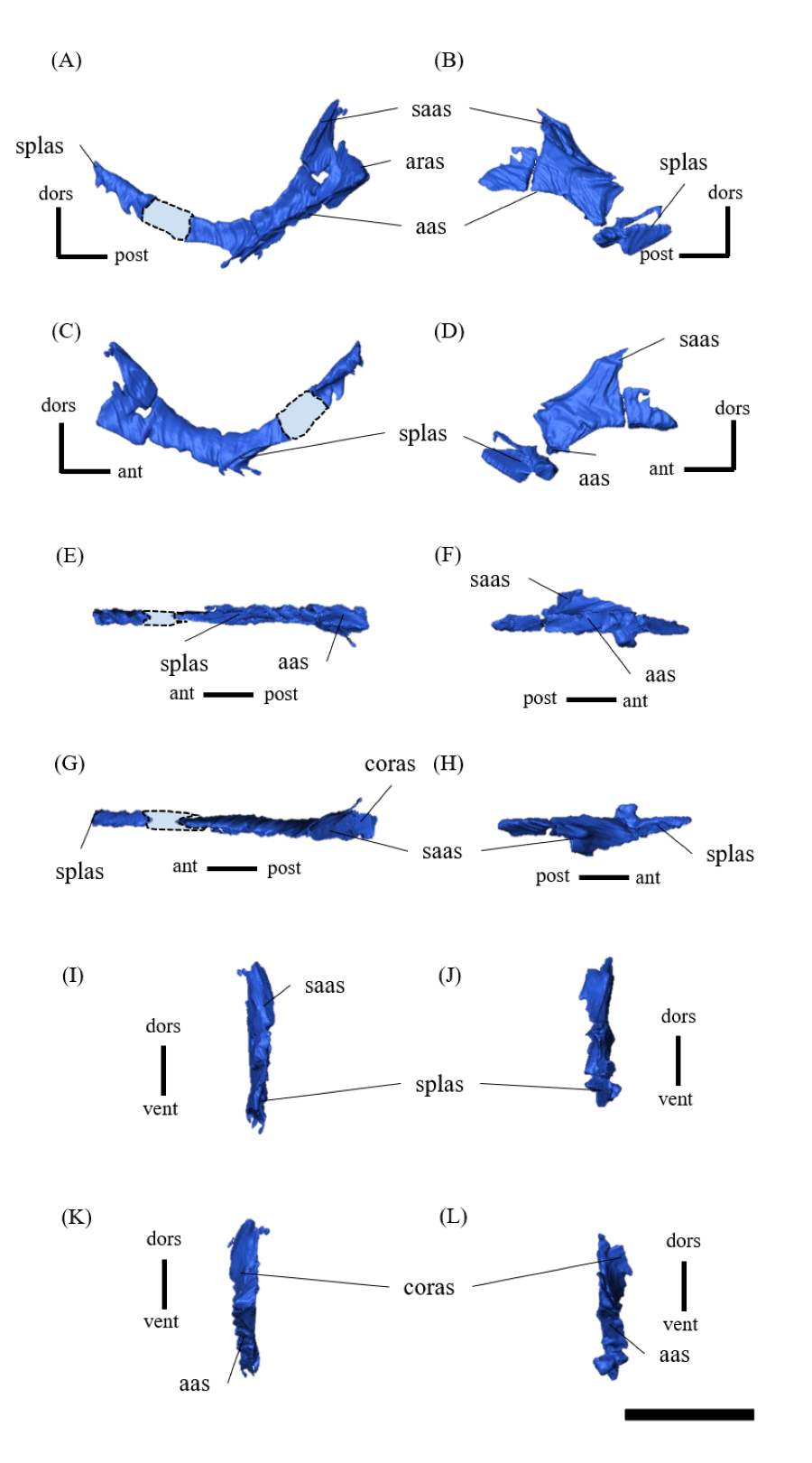

Supplement: Supplemental Information 33 — Black dotted line indicates the missing part. Abbreviations: aas, angular articular surface; aras, articular articular surface; coras, coronoid articular surface; saas, surangular articular surface; splas, splenial articular surface. Scale bar equals 20 mm. [file peerj-13-19547-s033.png]

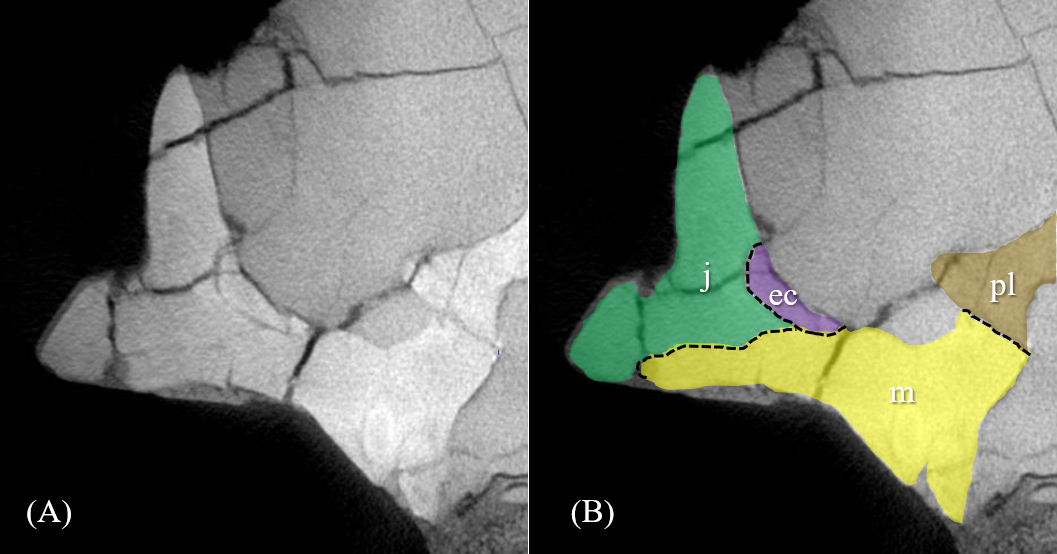

Supplement: Supplemental Information 34 — Black dotted lines in A indicate the suture lines. Abbreviations: ec, ectopterygoid; j, jugal; m, maxilla; pl, palatine. [file peerj-13-19547-s034.png]

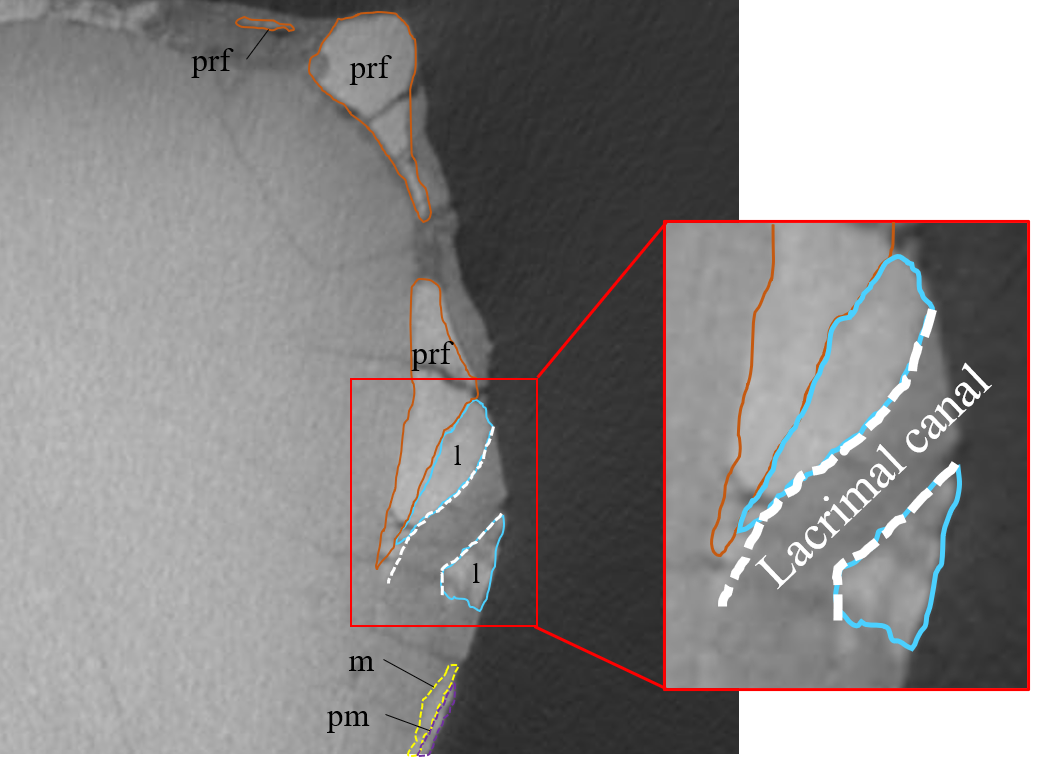

Supplement: Supplemental Information 35 — Each bone is segmented with colors. Abbreviations: l, lacrimal; m, maxilla; pm, premaxilla; prf, prefrontal. [file peerj-13-19547-s035.png]

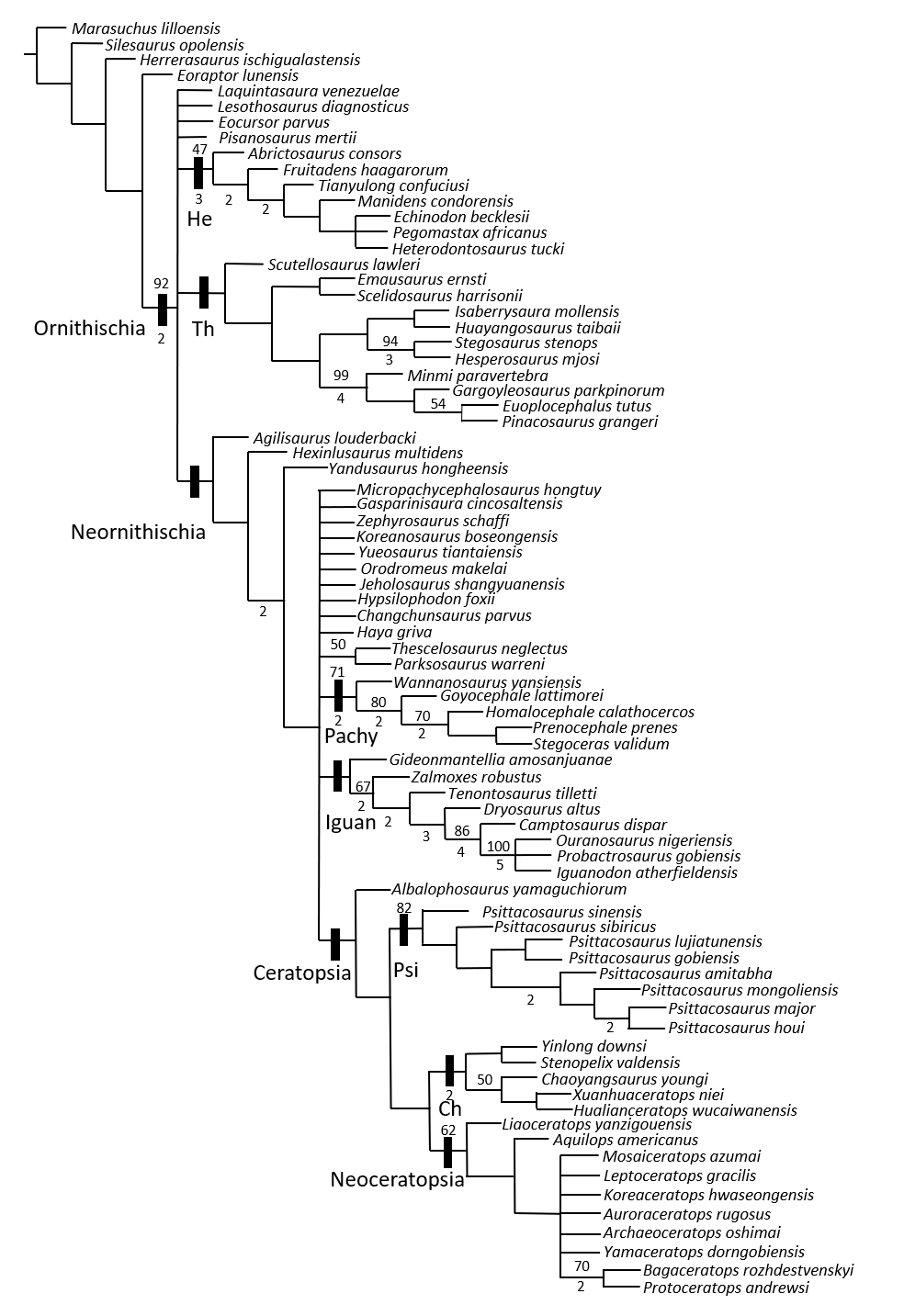

Supplement: Supplemental Information 36 — Values above and beneath each node indicate bootstrap and Bremer support, respectively (bootstrap values under 50 and Bremer support values equal 1 are not shown). Abbreviations: He, Heterodontosauridae; Th, Thyreophora; Pachy, Pachycephalosauria; Iguan, Iguanodontia; Psi, Psittacosaurus; Ch, Chaoyangsauridae. [file peerj-13-19547-s036.png]

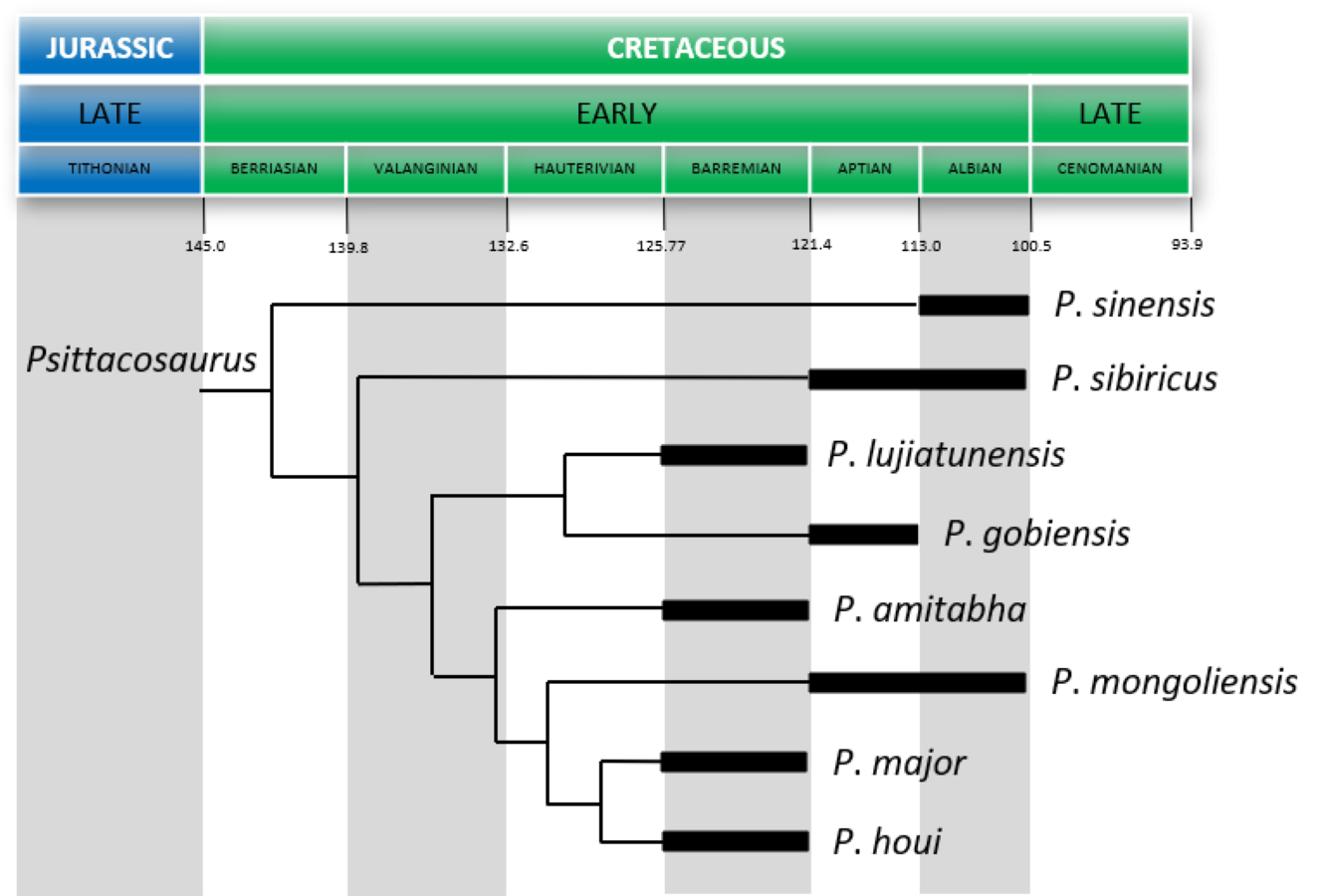

Supplement: Supplemental Information 37 — Each occurrence is based on Zhong et al. (2021) for P. houi , P. major and P. lujiatunensis , Napoli et al. (2019) for P. amitabha , and Sereno (2010) for P. sinensis , P. sibiricus , P. gobiensis and P. mongoliensis. [file peerj-13-19547-s037.png]

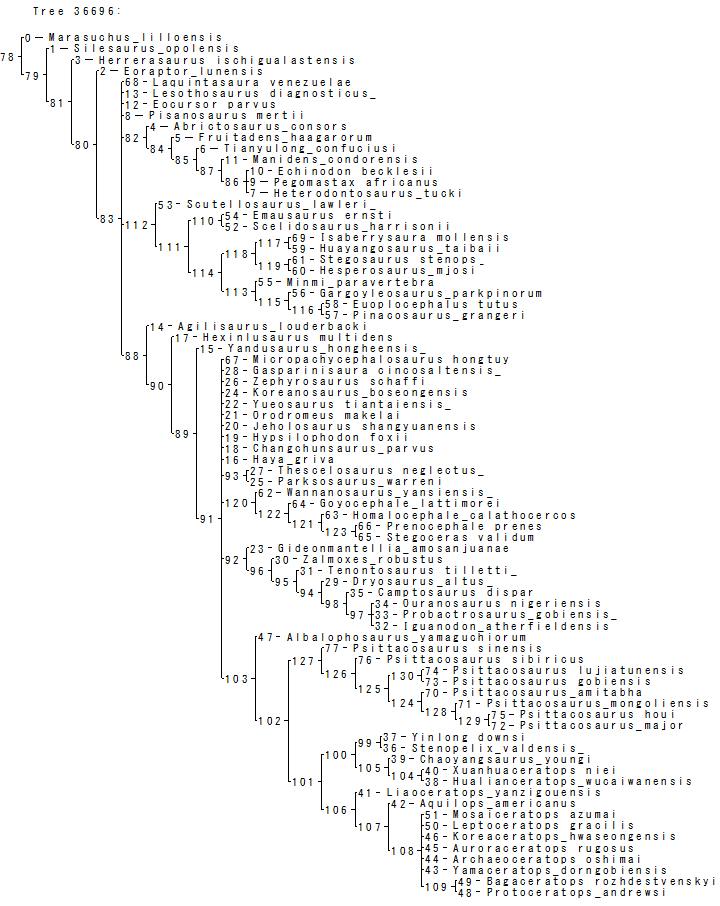

Supplement: Supplemental Information 38 [file peerj-13-19547-s038.png]
