# Supplementary material for: Psittacosaurus houi, a longer snouted psittacosaurid from the Lower Cretaceous Lujiatun Unit of Yixian Formation, China, with the synonymy of the unresolved genus Hongshanosaurus revisited
Source: PeerJ. 2025 Jul 8;13:e19547. doi: 10.7717/peerj.19547 (PMC12248233; doi:10.7717/peerj.19547)
Supplement: Supplemental Information 39 [file peerj-13-19547-s039.docx]

| **Species** | **Specimen number** | **Reference** |
| --- | --- | --- |
| *P*. *houi* | IVPP V12704 | You, Xu and Wang, 2003 |
|  | IVPP V12617 | You and Xu (2005); Tanoue, You and Dodson (2009); Taylor et al (2017); Bullar et al. (2019); Landi et al. (2021); Han et al. (2018) |
| *P*. *lujiatunensis* | ZMNH M8137 | Zhou et al. (2006); Sereno (2010) |
| *P*. *major* | LH PV1 | Sereno et al. (2007); Sereno (2010) |
|  | CAGS-IG-VD-004 | You, Tanoue and Dodson (2008) |
| *P*. *meileyingensis* | IVPP V7705 | Sereno et al. (1988); Sereno (2010) |
|  | CAGS-IG-V-330 | Sereno et al. (1988) |
| *P*. *mongoliensis* | AMNH 6254 | Osborn (1923); Sereno (2010) |
| *P*. *neimongoliensis* | IVPP 12-0888-2 | Russell and Zhao (1996); Sereno (2010) |
| *P*. *sibiricus* | PM TGU 16/0-15 | Averianov et al. (2007) |
|  | PM TGU 16/0-20 |  |
|  | PM TGU 16/0-30-39 |  |
|  | PM TGU 16/0-39 |  |
|  | PM TGU 16/1-11 |  |
|  | PM TGU 16/1-51 |  |
|  | PM TGU 16/1-136 |  |
|  | PM TGU 16/1-137 |  |
|  | PM TGU 16/1-166 |  |
|  | PM TGU 16/1-167 |  |
|  | PM TGU 16/1-175 |  |
|  | PM TGU 16/1-176 |  |
|  | PM TGU 16/1-179 |  |
|  | PM TGU 16/1-200 |  |
|  | PM TGU 16/1-201 |  |
|  | PM TGU 16/1-202 |  |
|  | PM TGU 16/1-203 |  |
|  | PM TGU 16/1-209 |  |
|  | PM TGU 16/1-216 |  |
|  | PM TGU 16/1-223 |  |
|  | PM TGU 16/1-228 |  |
|  | PM TGU 16/1-258 |  |
|  | PM TGU 16/1-271 |  |
|  | PM TGU 16/1-274 |  |
|  | PM TGU 16/1-276 |  |
|  | PM TGU 16/1-281 |  |
|  | PM TGU 16/1-283 |  |
|  | PM TGU 16/1-284 |  |
|  | KOKM 22985/2 | Podlesnov et al. (2023) |
| *P*. *sinensis* | IVPP V738 | Young (1958); Sereno (1990); Tanoue, You and Dodson (2009); Sereno (2010) |
|  | BNHM BPV149 | Chao (1962); Sereno (2010) |
| *P*. *xinjiangensis* | IVPP V7702 | Sereno and Chao (1988); Sereno (2010) |
| *P*. *gobiensis* | LH PV2 | Sereno, Zhao and Tan (2010) |
| *P*. *amitabha* | IGM 100/1132 | Napoli et al. (2019) |
