# Supplementary material for: Psittacosaurus houi, a longer snouted psittacosaurid from the Lower Cretaceous Lujiatun Unit of Yixian Formation, China, with the synonymy of the unresolved genus Hongshanosaurus revisited
Source: PeerJ. 2025 Jul 8;13:e19547. doi: 10.7717/peerj.19547 (PMC12248233; doi:10.7717/peerj.19547)
Supplement: Supplemental Information 40 [file peerj-13-19547-s040.docx]

| Element | Measurement taken | Dimension (mm) | |
| --- | --- | --- | --- |
|  |  | Left | Right |
| Skull | Skull length (The anterior-most extent of the rostral to the posterior-most extent of the quadrate condyle) | 121.1 | 122.5 |
|  | Basal skull length (The anterior-most extent of the rostral to the posterior-most end of the occipital condyle) | 136.8 | 139.1 |
|  | Preorbital length (The anterior-most extent of the rostral to the anterior-most margin of the orbit) | 60.8 | 57.4 |
|  | Skull width (Across the lateral-most extents of jugal horns) | 195.5 | |
|  | Skull height (The ventral-most dentary flange to the dorsal-most skull roof) | 115.6 | 125.1 |
| Rostral | Dorsoventral height | 36 | |
|  | Max. anteroposterior length | 16.8 | |
|  | Transverse width | 28.8 | |
| Premaxilla | Anteroposterior length | 54 | 47 |
|  | Dorsoventral height | 46.3 | 48.6 |
| Maxilla | Anteroposterior length | 55.2 | 54.8 |
|  | Dorsoventral height | 42.1 | 41.2 |
|  | Tooth row length | 29.8 | 31.6 |
| Nasal | Height | 21.2 | |
|  | Anteroposterior length (the anterior-most tip to the posterior-most edge) | 57.4 | |
| Jugal | Anteroposterior length | 90.4 | 86.1 |
|  | Dorsoventral height | 43 | 45.5 |
| Orbit | Anteroposterior length | 42.4 | 47.6 |
|  | Dorsoventral height | 48.8 | 45.4 |
| Quadratojugal | Anteroposterior length | 17.8 | 32.2 |
|  | Dorsoventral height | 35.8 | 29 |
| Quadrate | Anteroposterior length | 37.3 | 43.7 |
|  | Dorsoventral height | 67.3 | 68.8 |
| Squamosal | Anteroposterior length | 24.3 | 19.2 |
|  | Dorsoventral height | 18.1 | 21.1 |
| Parietal | Anteroposterior length | 43.1 | |
|  | Dorsoventral height | 22.1 | |
| basipterygoid process | From the anterior-most extent of the process to the root | 13.5 | 14.4 |
| body of basisphenoid | the notch between the basipterygoid processes to the basal tubera | 13.5 | |
| Foramen magnum | Transverse width | 18.3 | |
|  | Dorsoventral height | 13.2 | |
| Occipital condyle | Transverse width | 12.5 | |
|  | Dorsoventral height | 10.8 | |
| Lower jaw | Anteroposterior length | 117.5 | 118.3 |
|  | Dorsoventral height | 47 | 54.2 |
|  | Tooth row length | 32.1 | 32.9 |
