# Supplementary material for: Psittacosaurus houi, a longer snouted psittacosaurid from the Lower Cretaceous Lujiatun Unit of Yixian Formation, China, with the synonymy of the unresolved genus Hongshanosaurus revisited
Source: PeerJ. 2025 Jul 8;13:e19547. doi: 10.7717/peerj.19547 (PMC12248233; doi:10.7717/peerj.19547)
Supplement: Supplemental Information 41 — All characters are based on Sereno (2010) except the presence or absence of the lacrimal canal fenestra, which is considered as taphonomic artefact by Napoli et al. (2019) (see text). ✓ , present; -, absent; ?, undetermined. [file peerj-13-19547-s041.docx]

| **Characters** | ***Psittacosaurus*** | ***P*. *houi*** | |
| --- | --- | --- | --- |
|  |  |  |  |
|  |  | **ZMNH M12414** | **IVPP V12617** |
| preorbital length less than 40% of skull length | ✓ | - | - |
| external naris with ventral margin dorsal to that of the orbit | ✓ | ✓ | ✓ |
| nasal internarial process extending ventral to external naris | ✓ | ✓ | ✓ |
| rostral-nasal contact present | ✓ | ✓ | ✓ |
| premaxilla dorsolateral process maximum width subequal to dorsoventral orbital diameter | ✓ | ✓ | ✓ |
| premaxilla-prefrontal contact present | ✓ | ✓ | ? |
| premaxilla-jugal approximation or contact present | ✓ | ✓ | ✓ |
| maxillary fossa | ✓ | ✓ | ✓ |
| maxillary protuberance | ✓ | ✓ | ✓ |
| antorbital fenestra and fossa absent | ✓ | ✓ | ✓ |
| postorbital posterior process extends along the entire supratemporal bar | ✓ | ✓ | ✓ |
| end of squamosal anterior process situated on the dorsal aspect of the postorbital | ✓ | ✓ | ✓ |
| pterygoid with neomorphic palatal lamina forming the basal plate | ✓ | ✓ | ✓ |
| pterygoid with hypertrophied mandibular ramus | ✓ | ✓ | ✓ |
| medial quadrate condyle planar | ✓ | ✓ | ✓ |
| laterally divergent palpebral with transverse posterior margin | ✓ | ? | ? |
| predentary with very short, tongue-shaped ventral processes | ✓ | ✓ | ✓ |
| predentary with semicircular anterior margin | ✓ | ✓ | ✓ |
| dentary with ventral ridge or flange | ✓ | ✓ | ✓ |
| articular with planar surface for quadrate condyles | ✓ | ✓ | ✓ |
| dentary teeth with bulbous cone-shaped primary ridge with secondary ridging | ✓ | ? | ✓ |
