# Supplementary material for: Psittacosaurus houi, a longer snouted psittacosaurid from the Lower Cretaceous Lujiatun Unit of Yixian Formation, China, with the synonymy of the unresolved genus Hongshanosaurus revisited
Source: PeerJ. 2025 Jul 8;13:e19547. doi: 10.7717/peerj.19547 (PMC12248233; doi:10.7717/peerj.19547)
Supplement: Supplemental Information 42 — ✓ , present; -, absent. [file peerj-13-19547-s042.docx]

| **Characters** | **Citation** | ***P*. *houi*** | | ***P*. *lujiatunensis*** | ***P*. *major*** |
| --- | --- | --- | --- | --- | --- |
|  |  |  |  |  |  |
|  |  | **ZMNH M12414** | **IVPP V12617** |  |  |
| narrow prefrontal-premaxilla contact | in this study | ✓ | ✓ | - | - |
| higher ventral-most margin of the premaxilla raised above the maxillary tooth row | in this study | ✓ | ✓ | - | - |
| axes of maxillary tooth row and dorsal process of jugal oriented an angle of about 135 degrees in lateral view | in this study | ✓ | ✓ | - | - |
| subtriangular supraoccipital widest at its ventral margin | in this study | ✓ | ✓ | - | - |
| long jugal bar of the postorbital process nearly twice as a temporal bar of the bone | in this study | ✓ | ✓ | - | - |
| long preorbital region reaching about one half of the skull length | in this study | ✓ | ✓ | - | - |
| posterodorsally-elongated laterotemporal fenestra oriented at an angle of about 45 degrees in lateral view | in this study | ✓ | ✓ | - | - |
| posterior margin of the parietal nearly linear perpendicular to the sagittal crest with no indentation on the midline | in this study | ✓ | ✓ | - | - |
| anterior margin of rostral and nasal gently sloped posterodorsally at 30 degree | in this study | ✓ | ✓ | - | - |
| height of the retroarticular process approximately at the same level as the dentary tooth row | in this study | ✓ | ✓ | - | - |
| proportion of the width across the anterior margins to that across the posterior margins of the maxillary tooth rows more than 45% in ventral view | Han et al., 2018 | ✓ | ✓ | ✓ | - |
| triangular, or subtriangular, shape of the maxillary fossa | Han et al., 2018 | ✓ | ✓ | ✓ | - |
| infratemporal ramus of the jugal forming the anteroventral margin of the infratemporal fenestra, without expanding dorsally to form a part of the posterior margin | Han et al., 2018 | ✓ | ✓ | ✓ | - |
| elongate basipterygoid processes subequal in length to the body of the basisphenoid as measured from the notch between the processes to the basal tubera | Sereno, 2010; Hedrick and Dodson, 2013 | ✓ | ✓ | - | ✓ |
| external naris lying above the maxilla | Han et al., 2018 | ✓ | ✓ | - | ✓ |
| position of the maxillary fossa lying anterior to the orbit | Han et al., 2018 | ✓ | ✓ | - | ✓ |
| strongly curved ventral margin of the dentary | Han et al., 2018 | ✓ | ✓ | - | ✓ |
