# Supplementary material for: Psittacosaurus houi, a longer snouted psittacosaurid from the Lower Cretaceous Lujiatun Unit of Yixian Formation, China, with the synonymy of the unresolved genus Hongshanosaurus revisited
Source: PeerJ. 2025 Jul 8;13:e19547. doi: 10.7717/peerj.19547 (PMC12248233; doi:10.7717/peerj.19547)
Supplement: Supplemental Information 43 — All characters are based on Sereno (2010), except for P. gobiensis (Sereno, 2010 and P. amitabha (Napoli et al., 2019). P. lujiatunensis and P. major were excluded because the referred characters were shown to be invalid (Hedrick & Dodson, 2013). ✓ , present in ZMNH M12414; -, absent in ZMNH M12414; ?, undetermined. [file peerj-13-19547-s043.docx]

| **Species** | **Characters** | ***P*. *houi*** | |
| --- | --- | --- | --- |
|  |  | **ZMNH M12414** | **IVPP V12617** |
| *P*. *meileyingensis* | preorbital length only approximately 30% of skull length | - | - |
|  | subtriangular orbit with acute ventral corner | - | - |
|  | rugose quadratojugal eminence | - | - |
| *P*. *mongoliensis* | a raised lip on the orbital margin of the prefrontal | - | - |
|  | transverse expansion of the distal end of the ischial blade to approximately twice its width at mid-shaft | ? | ? |
| *P*. *neimongoliensis* | posterior end of the nasal contacting its opposite in the midline (not separated by the frontal) | ? | - |
|  | frontal interorbital width approximately 30% of frontal length | - | - |
|  | postorbital extending along the margin of the orbit (rather than inset from the margin by the frontal in dorsal view) | - | - |
| *P*. *sibiricus* | laterotemporal fenestra subequal in maximum height and anteroposterior length | - | - |
|  | postorbital ventral process with subvertical orientation set at an angle of approximately 95°to the posterior process | - | - |
|  | postorbital with small dorsal horn | - | - |
|  | enlarged palpebral subequal in transverse width to the adjacent skull roof | ? | ? |
|  | palpebral posterior margin nearly straight and angled anterolaterally | ? | ? |
|  | predentary dorsoventrally compressed with a wedge-shaped profile with external margins set at approximately 30° | - | - |
|  | angular with arcuate ventral extension of the dentary flange | - | - |
|  | angular process projecting laterally at posterior end of the ventral flange of the mandible | - | - |
|  | 14 dorsal vertebrae | ? | ? |
| *P*. *sinensis* | pendant rostrum that positions the ventral edge of the rostral bone below the level of the maxillary tooth row | - | - |
|  | anteroventral processes of the nasal separated in the midline by a narrow gap | - | - |
|  | short lower jaw that positions the anterior margin of the predentary in opposition to the premaxilla rather than the rostral | - | - |
|  | posteriorly flaring skull roof with postorbital-squamosal bars diverging at an angle of approximately 30° | ✓ | ✓ |
|  | absence of the maxillary fossa | - | - |
|  | absence of the maxillary protuberance | - | - |
|  | vertically elongate horn on the postorbital bar split between jugal and postorbital | - | - |
|  | frontal participation in the supratemporal fossa | ✓ | ✓ |
|  | ectopterygoid far removed from postpalatine foramen by broad maxilla-pterygoid contact | - | - |
|  | internal mandibular fenestra reduced to a foramen | ? | ? |
|  | absence of ossified tendons | ? | ? |
|  | prepubic and postpubic processes transversely broad throughout their length (transversely wider than dorsoventrally tall) | ? | ? |
|  | prepubic process projecting anteriorly as far as the preacetabular process of the ilium | ? | ? |
| *P*. *xinjiangensis* | hook-shaped palpebral with V-shaped posterior margin | ? | ? |
|  | dentary teeth with as many 21 denticles in the posterior center of the tooth row | ? | - |
|  | ossified tendons extending into mid-caudal vertebrae | ? | ? |
|  | narrow iliac postacetabular process with height at midlength less than 25% of the length of the process | ? | ? |
| *P*. *gobiensis* | pyramidal horn on the postorbital bar composed almost entirely of the postorbital | - | - |
|  | postorbital–jugal fossa | - | - |
|  | minimum width of the postorbital bar approximately 50 per cent the width of the base of the process | - | - |
|  | retroarticular process deflected posteromedially at an angle of 40 degrees from the axis of the mandible | - | - |
|  | thin and restricted enamel on medial and lateral aspects of the maxillary and dentary crowns, respectively | ? | ? |
| *P*. *amitabha* | a relatively longer snout than *Psittacosaurus* *mongoliensis*, with a less steeply inclined anterior rostronasal margin | ✓ | ✓ |
|  | a cranium dorsally convex rather than flat | - | - |
|  | a subtemporal length less than 40% of total skull length | ? | ? |
|  | 5 premaxillary foramina arranged in an arc | - | ? |
|  | posterior lamina of the maxilla cupped around the toothrow | - | - |
|  | an antorbital fossa as long as tall | - | - |
|  | a palpebral with a well-developed posterior tonguelike process | ? | ? |
