# Supplementary material for: Psittacosaurus houi, a longer snouted psittacosaurid from the Lower Cretaceous Lujiatun Unit of Yixian Formation, China, with the synonymy of the unresolved genus Hongshanosaurus revisited
Source: PeerJ. 2025 Jul 8;13:e19547. doi: 10.7717/peerj.19547 (PMC12248233; doi:10.7717/peerj.19547)
Supplement: Supplemental Information 44 [file peerj-13-19547-s044.docx]

Marasuchus_lilloensis :

No autapomorphies

Silesaurus_opolensis :

Char. 205: 0 --> 1

Char. 207: 0 --> 1

Char. 220: 0 --> 1

Char. 224: 0 --> 1

Char. 354: 0 --> 1

Eoraptor_lunensis :

Char. 21: 0 --> 1

Char. 53: 0 --> 2

Char. 64: 2 --> 0

Char. 94: 1 --> 0

Char. 98: 0 --> 1

Char. 182: 0 --> 1

Char. 276: 0 --> 1

Char. 320: 1 --> 0

Char. 323: 0 --> 1

Char. 329: 0 --> 1

Char. 331: 0 --> 1

Herrerasaurus_ischigualastensis :

Char. 20: 1 --> 0

Char. 64: 2 --> 1

Char. 89: 1 --> 0

Char. 102: 1 --> 0

Char. 125: 0 --> 2

Char. 274: 0 --> 2

Char. 278: 0 --> 1

Abrictosaurus_consors :

Char. 192: 0 --> 1

Char. 213: 1 --> 0

Char. 221: 0 --> 1

Char. 226: 0 --> 1

Char. 307: 0 --> 1

Char. 319: 0 --> 1

Fruitadens_haagarorum :

Char. 197: 0 --> 1

Tianyulong_confuciusi :

Char. 155: 0 --> 1

Char. 190: 0 --> 1

Char. 194: 0 --> 1

Char. 196: 3 --> 4

Char. 216: 1 --> 0

Char. 257: 0 --> 1

Heterodontosaurus_tucki :

Char. 226: 0 --> 1

Pisanosaurus_mertii :

Char. 42: 0 --> 1

Char. 169: 0 --> 1

Char. 192: 0 --> 1

Char. 207: 0 --> 1

Char. 214: 1 --> 0

Char. 360: 1 --> 0

Char. 361: 2 --> 1

Char. 367: 1 --> 0

Char. 373: 1 --> 0

Pegomastax_africanus :

Char. 167: 0 --> 1

Char. 177: 1 --> 0

Char. 211: 0 --> 1

Echinodon_becklesii :

Char. 164: 0 --> 1

Char. 165: 1 --> 0

Char. 222: 1 --> 0

Manidens_condorensis :

Char. 180: 0 --> 1

Eocursor_parvus_ :

Char. 185: 0 --> 1

Char. 268: 1 --> 0

Char. 269: 0 --> 1

Char. 322: 0 --> 1

Char. 346: 0 --> 1

Lesothosaurus_diagnosticus_ :

Char. 44: 0 --> 1

Char. 49: 0 --> 1

Char. 51: 0 --> 1

Char. 102: 1 --> 0

Char. 129: 1 --> 0

Char. 169: 0 --> 1

Char. 185: 0 --> 1

Char. 192: 0 --> 1

Char. 257: 0 --> 1

Char. 274: 0 --> 2

Char. 276: 0 --> 1

Char. 282: 1 --> 0

Char. 306: 2 --> 1

Char. 333: 0 --> 1

Char. 335: 1 --> 0

Agilisaurus_louderbacki :

Char. 12: 0 --> 1

Char. 37: 1 --> 0

Char. 47: 0 --> 1

Char. 59: 0 --> 2

Char. 120: 0 --> 1

Char. 135: 0 --> 1

Char. 192: 0 --> 1

Char. 198: 0 --> 1

Char. 201: 0 --> 2

Char. 203: 1 --> 0

Char. 215: 0 --> 2

Char. 276: 0 --> 1

Char. 277: 0 --> 2

Char. 328: 0 --> 1

Char. 335: 1 --> 0

Yandusaurus_hongheensis_ :

Char. 241: 0 --> 1

Char. 277: 0 --> 2

Char. 280: 0 --> 1

Haya_griva :

Char. 49: 0 --> 1

Char. 71: 0 --> 1

Char. 79: 0 --> 1

Char. 164: 0 --> 1

Char. 182: 1 --> 0

Char. 238: 1 --> 0

Char. 258: 0 --> 1

Char. 335: 1 --> 0

Char. 344: 1 --> 0

Char. 347: 0 --> 1

Hexinlusaurus_multidens_ :

Char. 71: 0 --> 1

Char. 102: 1 --> 0

Char. 306: 2 --> 1

Changchunsaurus_parvus :

Char. 38: 0 --> 2

Char. 40: 2 --> 1

Char. 57: 0 --> 1

Char. 58: 0 --> 1

Char. 74: 0 --> 1

Char. 164: 0 --> 1

Char. 169: 0 --> 1

Char. 192: 0 --> 1

Char. 203: 0 --> 1

Char. 232: 0 --> 1

Hypsilophodon_foxii_ :

Char. 26: 1 --> 0

Char. 36: 0 --> 1

Char. 49: 0 --> 1

Char. 79: 0 --> 1

Char. 89: 0 --> 2

Char. 128: 0 --> 1

Char. 135: 0 --> 1

Char. 192: 0 --> 1

Char. 208: 1 --> 0

Char. 210: 0 --> 1

Char. 264: 0 --> 1

Jeholosaurus_shangyuanensis :

Char. 10: 0 --> 1

Char. 58: 0 --> 1

Char. 79: 0 --> 1

Char. 144: 0 --> 1

Char. 160: 1 --> 0

Char. 169: 0 --> 1

Char. 182: 1 --> 0

Char. 196: 1 --> 0

Char. 232: 0 --> 1

Char. 332: 0 --> 1

Char. 335: 1 --> 0

Char. 347: 0 --> 1

Char. 371: 0 --> 1

Orodromeus_makelai :

Char. 34: 0 --> 1

Char. 41: 0 --> 1

Char. 57: 0 --> 1

Char. 71: 0 --> 1

Char. 120: 0 --> 1

Char. 207: 1 --> 0

Char. 306: 2 --> 1

Yueosaurus_tiantaiensis_ :

No autapomorphies

Gideonmantellia_amosanjuanae :

Char. 306: 2 --> 1

Char. 318: 1 --> 0

Koreanosaurus_boseongensis :

Char. 234: 1 --> 0

Char. 268: 1 --> 2

Parksosaurus_warreni :

Char. 192: 0 --> 1

Char. 205: 1 --> 2

Char. 238: 1 --> 2

Char. 253: 1 --> 3

Zephyrosaurus_schaffi_ :

Char. 41: 0 --> 1

Char. 49: 0 --> 1

Char. 50: 0 --> 1

Char. 57: 0 --> 1

Char. 71: 0 --> 1

Char. 135: 0 --> 1

Char. 203: 0 --> 1

Thescelosaurus_neglectus_ :

Char. 37: 1 --> 0

Char. 47: 0 --> 1

Char. 95: 1 --> 0

Char. 214: 1 --> 0

Char. 220: 1 --> 0

Char. 222: 1 --> 0

Char. 349: 1 --> 0

Gasparinisaura_cincosaltensis_ :

Char. 34: 0 --> 1

Char. 37: 1 --> 0

Char. 81: 1 --> 0

Char. 87: 0 --> 1

Char. 192: 0 --> 1

Char. 209: 0 --> 1

Char. 243: 3 --> 2

Char. 253: 1 --> 3

Char. 306: 2 --> 3

Char. 320: 1 --> 0

Char. 371: 0 --> 1

Char. 377: 0 --> 1

Dryosaurus_altus_ :

Char. 2: 0 --> 1

Char. 14: 0 --> 1

Char. 25: 1 --> 0

Char. 32: 0 --> 1

Char. 39: 0 --> 1

Char. 44: 0 --> 1

Char. 47: 0 --> 1

Char. 176: 1 --> 0

Char. 182: 1 --> 0

Char. 235: 1 --> 0

Char. 238: 2 --> 1

Char. 348: 1 --> 0

Char. 357: 1 --> 0

Zalmoxes_robustus :

Char. 45: 0 --> 1

Char. 59: 0 --> 1

Char. 70: 0 --> 1

Char. 130: 0 --> 1

Char. 163: 1 --> 0

Char. 211: 0 --> 1

Char. 241: 0 --> 1

Char. 269: 0 --> 1

Char. 273: 1 --> 0

Char. 313: 0 --> 1

Char. 314: 0 --> 1

Char. 325: 0 --> 1

Char. 328: 0 --> 1

Char. 333: 1 --> 0

Tenontosaurus_tilletti_ :

Char. 49: 0 --> 1

Char. 79: 0 --> 1

Char. 81: 1 --> 0

Char. 133: 2 --> 1

Char. 160: 1 --> 0

Char. 227: 1 --> 2

Char. 231: 0 --> 1

Char. 236: 1 --> 0

Char. 243: 3 --> 2

Char. 249: 0 --> 1

Char. 306: 2 --> 3

Iguanodon_atherfieldensis :

Char. 126: 1 --> 2

Char. 130: 0 --> 1

Char. 182: 1 --> 0

Char. 211: 0 --> 1

Char. 280: 0 --> 1

Char. 294: 1 --> 0

Char. 328: 0 --> 1

Char. 368: 1 --> 0

Probactrosaurus_gobiensis_ :

Char. 226: 1 --> 2

Ouranosaurus_nigeriensis_ :

Char. 94: 0 --> 1

Char. 126: 1 --> 0

Char. 250: 0 --> 1

Camptosaurus_dispar :

Char. 13: 0 --> 1

Char. 90: 0 --> 1

Char. 125: 2 --> 0

Char. 243: 3 --> 2

Char. 253: 1 --> 3

Char. 328: 0 --> 1

Stenopelix_valdensis_ :

Char. 311: 1 --> 2

Char. 313: 0 --> 1

Char. 314: 0 --> 1

Char. 315: 1 --> 0

Char. 328: 1 --> 2

Char. 344: 2 --> 0

Char. 349: 1 --> 0

Yinlong_downsi :

No autapomorphies

Hualianceratops_wucaiwanensis :

Char. 85: 1 --> 0

Char. 93: 2 --> 0

Chaoyangsaurus_youngi :

Char. 43: 0 --> 1

Char. 156: 1 --> 0

Xuanhuaceratops_niei :

Char. 167: 0 --> 2

Liaoceratops_yanzigouensis :

Char. 17: 0 --> 1

Char. 182: 1 --> 0

Char. 204: 0 --> 1

Char. 222: 1 --> 0

Aquilops_americanus :

Char. 10: 0 --> 1

Char. 35: 1 --> 0

Char. 51: 0 --> 1

Char. 212: 0 --> 1

Yamaceratops_dorngobiensis :

Char. 53: 2 --> 0

Char. 58: 1 --> 0

Char. 158: 1 --> 0

Char. 167: 0 --> 2

Char. 169: 1 --> 0

Char. 171: 0 --> 1

Char. 183: 1 --> 0

Char. 202: 1 --> 0

Char. 222: 1 --> 0

Char. 307: 0 --> 1

Archaeoceratops_oshimai :

Char. 10: 0 --> 1

Char. 34: 1 --> 0

Char. 56: 1 --> 0

Char. 67: 0 --> 2

Char. 86: 1 --> 0

Char. 164: 1 --> 0

Char. 169: 1 --> 0

Char. 170: 0 --> 1

Char. 226: 2 --> 1

Char. 241: 1 --> 0

Char. 307: 0 --> 1

Char. 314: 0 --> 1

Char. 328: 1 --> 0

Char. 375: 1 --> 0

Auroraceratops_rugosus :

Char. 10: 0 --> 1

Char. 31: 2 --> 1

Char. 67: 0 --> 2

Char. 158: 1 --> 0

Char. 164: 1 --> 0

Char. 170: 0 --> 1

Char. 180: 1 --> 0

Char. 238: 0 --> 1

Char. 239: 0 --> 1

Char. 313: 0 --> 1

Char. 314: 0 --> 1

Char. 329: 0 --> 1

Char. 357: 0 --> 1

Koreaceratops_hwaseongensis :

Char. 328: 1 --> 0

Char. 329: 0 --> 1

Albalophosaurus_yamaguchiorum :

Char. 42: 1 --> 0

Char. 175: 1 --> 0

Protoceratops_andrewsi :

Char. 9: 0 --> 1

Char. 64: 0 --> 2

Char. 171: 0 --> 1

Bagaceratops_rozhdestvenskyi :

Char. 58: 1 --> 0

Char. 114: 2 --> 1

Leptoceratops_gracilis_ :

Char. 54: 0 --> 1

Char. 101: 1 --> 0

Char. 106: 1 --> 0

Char. 167: 0 --> 2

Char. 203: 0 --> 1

Char. 204: 0 --> 1

Char. 226: 2 --> 1

Char. 253: 1 --> 0

Char. 268: 1 --> 0

Char. 274: 0 --> 1

Char. 292: 0 --> 1

Char. 294: 0 --> 1

Char. 348: 0 --> 1

Char. 358: 0 --> 1

Mosaiceratops_azumai :

Char. 6: 1 --> 0

Char. 24: 0 --> 1

Char. 28: 0 --> 1

Char. 56: 1 --> 0

Char. 59: 2 --> 0

Char. 61: 0 --> 1

Char. 69: 1 --> 0

Char. 73: 0 --> 1

Char. 74: 1 --> 0

Char. 133: 1 --> 2

Char. 180: 1 --> 0

Char. 329: 0 --> 1

Scelidosaurus_harrisonii :

Char. 62: 0 --> 1

Char. 180: 0 --> 1

Char. 193: 0 --> 1

Scutellosaurus_lawleri_ :

Char. 204: 1 --> 0

Char. 234: 1 --> 0

Char. 249: 0 --> 1

Emausaurus_ernsti_ :

Char. 64: 2 --> 1

Minmi_paravertebra :

No autapomorphies

Gargoyleosaurus_parkpinorum :

No autapomorphies

Pinacosaurus_grangeri :

Char. 203: 12 --> 0

Euoplocephalus_tutus :

No autapomorphies

Huayangosaurus_taibaii :

Char. 74: 0 --> 1

Hesperosaurus_mjosi :

Char. 228: 1 --> 0

Char. 238: 12 --> 0

Stegosaurus_stenops_ :

Char. 25: 0 --> 1

Char. 94: 1 --> 0

Char. 213: 1 --> 0

Char. 236: 1 --> 0

Char. 343: 0 --> 1

Wannanosaurus_yansiensis_ :

Char. 102: 1 --> 0

Char. 206: 0 --> 1

Char. 282: 1 --> 0

Char. 357: 0 --> 1

Homalocephale_calathocercos :

Char. 128: 0 --> 1

Goyocephale_lattimorei :

Char. 243: 3 --> 1

Stegoceras_validum :

Char. 31: 1 --> 0

Char. 90: 1 --> 0

Char. 143: 1 --> 2

Prenocephale_prenes :

Char. 2: 1 --> 0

Char. 201: 0 --> 1

Micropachycephalosaurus_hongtuy :

Char. 85: 0 --> 1

Char. 93: 0 --> 1

Char. 207: 1 --> 0

Char. 213: 1 --> 0

Laquintasaura_venezuelae :

Char. 205: 1 --> 0

Char. 221: 0 --> 1

Char. 253: 1 --> 0

Char. 330: 0 --> 1

Char. 365: 0 --> 1

Isaberrysaura_mollensis :

Char. 31: 1 --> 0

Char. 47: 2 --> 1

Char. 52: 0 --> 1

Char. 59: 2 --> 0

Char. 66: 0 --> 1

Char. 202: 0 --> 1

Psittacosaurus_amitabha :

Char. 2: 1 --> 0

Char. 35: 0 --> 1

Char. 64: 1 --> 0

Char. 105: 1 --> 0

Char. 155: 1 --> 0

Char. 164: 1 --> 0

Char. 256: 1 --> 0

Psittacosaurus_mongoliensis :

Char. 54: 0 --> 1

Char. 84: 1 --> 0

Char. 125: 1 --> 0

Char. 166: 0 --> 1

Char. 194: 2 --> 1

Psittacosaurus_major :

Char. 2: 1 --> 2

Char. 34: 0 --> 1

Char. 65: 0 --> 1

Char. 102: 1 --> 0

Char. 122: 1 --> 0

Char. 126: 0 --> 1

Char. 175: 1 --> 0

Char. 184: 1 --> 0

Psittacosaurus_gobiensis :

Char. 30: 0 --> 1

Char. 82: 0 --> 1

Char. 93: 2 --> 0

Char. 155: 1 --> 0

Char. 167: 0 --> 1

Char. 186: 1 --> 0

Char. 189: 0 --> 1

Psittacosaurus_lujiatunensis :

Char. 3: 0 --> 1

Char. 81: 1 --> 0

Char. 83: 0 --> 1

Char. 160: 0 --> 1

Char. 211: 0 --> 1

Psittacosaurus_houi :

Char. 2: 1 --> 0

Char. 96: 0 --> 1

Char. 125: 1 --> 2

Char. 129: 1 --> 0

Char. 160: 0 --> 1

Psittacosaurus_sibiricus :

Char. 49: 1 --> 0

Char. 143: 1 --> 0

Char. 144: 0 --> 1

Char. 167: 0 --> 1

Char. 175: 1 --> 0

Char. 222: 1 --> 2

Char. 239: 0 --> 1

Char. 242: 0 --> 1

Char. 243: 3 --> 2

Char. 253: 1 --> 0

Char. 282: 1 --> 0

Char. 320: 12 --> 0

Char. 324: 1 --> 0

Char. 349: 1 --> 0

Psittacosaurus_sinensis :

Char. 3: 0 --> 1

Char. 12: 0 --> 2

Char. 180: 1 --> 0

Char. 192: 0 --> 1

Char. 343: 0 --> 1

Node 79 :

No synapomorphies

Node 80 :

Char. 13: 0 --> 1

Char. 219: 0 --> 1

Char. 234: 0 --> 1

Char. 236: 0 --> 1

Char. 237: 0 --> 1

Char. 305: 1 --> 0

Char. 306: 1 --> 2

Char. 332: 0 --> 1

Char. 349: 0 --> 1

Char. 361: 0 --> 1

Char. 364: 0 --> 1

Char. 374: 0 --> 1

Node 81 :

Char. 203: 0 --> 1

Char. 270: 1 --> 0

Char. 282: 0 --> 1

Char. 306: 0 --> 1

Char. 362: 0 --> 1

Node 82 :

Char. 12: 0 --> 1

Char. 16: 0 --> 1

Char. 78: 0 --> 1

Char. 175: 0 --> 1

Char. 196: 01 --> 3

Char. 203: 1 --> 0

Char. 204: 1 --> 0

Char. 216: 0 --> 1

Char. 218: 1 --> 0

Char. 286: 0 --> 1

Char. 293: 0 --> 1

Char. 322: 0 --> 1

Char. 323: 0 --> 1

Char. 363: 0 --> 1

Node 83 :

Char. 2: 0 --> 1

Char. 19: 0 --> 1

Char. 34: 1 --> 0

Char. 37: 0 --> 1

Char. 40: 0 --> 2

Char. 46: 0 --> 1

Char. 103: 0 --> 1

Char. 154: 0 --> 1

Char. 181: 0 --> 1

Char. 196: 2 --> 01

Char. 205: 0 --> 1

Char. 213: 0 --> 1

Char. 214: 0 --> 1

Char. 217: 0 --> 1

Char. 218: 0 --> 1

Char. 220: 0 --> 1

Char. 223: 0 --> 1

Char. 224: 0 --> 1

Char. 243: 1 --> 2

Char. 253: 0 --> 1

Char. 256: 0 --> 1

Char. 291: 1 --> 0

Char. 302: 0 --> 1

Char. 308: 0 --> 1

Char. 311: 0 --> 2

Char. 336: 0 --> 1

Char. 337: 0 --> 1

Char. 342: 0 --> 1

Char. 360: 0 --> 1

Char. 361: 1 --> 2

Char. 367: 0 --> 1

Char. 371: 0 --> 1

Node 84 :

Char. 215: 0 --> 1

Char. 354: 0 --> 1

Char. 356: 0 --> 1

Node 85 :

Char. 165: 0 --> 1

Char. 201: 0 --> 1

Char. 202: 0 --> 1

Node 86 :

Char. 207: 0 --> 1

Char. 210: 0 --> 1

Char. 218: 0 --> 1

Node 87 :

Char. 42: 0 --> 1

Char. 221: 0 --> 1

Node 88 :

Char. 26: 0 --> 1

Char. 42: 0 --> 1

Char. 51: 0 --> 1

Char. 81: 0 --> 1

Char. 90: 0 --> 1

Char. 93: 1 --> 0

Char. 94: 1 --> 0

Char. 128: 1 --> 0

Char. 174: 0 --> 1

Char. 175: 0 --> 1

Char. 180: 0 --> 1

Char. 273: 0 --> 1

Char. 333: 0 --> 1

Char. 344: 0 --> 1

Char. 345: 0 --> 1

Node 89 :

Char. 226: 0 --> 1

Char. 352: 0 --> 1

Char. 353: 1 --> 2

Node 90 :

Char. 72: 0 --> 1

Char. 207: 0 --> 1

Char. 323: 0 --> 1

Char. 332: 1 --> 0

Char. 350: 0 --> 1

Node 91 :

Char. 31: 0 --> 1

Char. 203: 1 --> 0

Char. 354: 0 --> 1

Node 92 :

Char. 343: 0 --> 1

Char. 354: 1 --> 0

Node 93 :

Char. 2: 1 --> 0

Char. 26: 1 --> 0

Char. 34: 0 --> 1

Char. 164: 0 --> 1

Char. 203: 0 --> 1

Char. 204: 0 --> 1

Char. 234: 1 --> 0

Char. 264: 0 --> 1

Char. 268: 1 --> 2

Char. 277: 0 --> 2

Char. 344: 1 --> 4

Char. 357: 0 --> 1

Node 94 :

Char. 10: 0 --> 1

Char. 203: 0 --> 1

Char. 209: 0 --> 1

Char. 212: 0 --> 1

Char. 306: 2 --> 1

Char. 320: 1 --> 0

Char. 329: 0 --> 1

Char. 371: 0 --> 1

Node 95 :

Char. 21: 0 --> 1

Char. 90: 1 --> 0

Char. 124: 0 --> 1

Char. 157: 0 --> 1

Char. 164: 0 --> 1

Char. 208: 1 --> 0

Char. 214: 1 --> 0

Char. 331: 0 --> 1

Node 96 :

Char. 348: 0 --> 1

Char. 349: 1 --> 0

Char. 357: 0 --> 1

Char. 358: 0 --> 1

Node 97 :

Char. 31: 1 --> 2

Char. 55: 0 --> 1

Char. 59: 0 --> 1

Char. 80: 0 --> 1

Char. 123: 0 --> 1

Char. 124: 1 --> 2

Char. 164: 1 --> 2

Char. 179: 0 --> 1

Char. 203: 1 --> 2

Char. 204: 1 --> 2

Char. 225: 0 --> 1

Char. 227: 1 --> 2

Char. 273: 1 --> 0

Char. 292: 1 --> 2

Char. 293: 0 --> 1

Char. 299: 0 --> 1

Char. 300: 0 --> 1

Char. 319: 0 --> 1

Char. 338: 0 --> 1

Char. 343: 1 --> 2

Char. 375: 1 --> 2

Node 98 :

Char. 51: 1 --> 0

Char. 135: 1 --> 2

Char. 204: 0 --> 1

Char. 222: 1 --> 2

Char. 241: 0 --> 1

Char. 247: 0 --> 2

Char. 258: 0 --> 1

Char. 283: 0 --> 1

Char. 285: 0 --> 1

Char. 288: 0 --> 1

Char. 303: 0 --> 1

Char. 334: 0 --> 1

Char. 347: 0 --> 1

Char. 369: 1 --> 0

Node 99 :

Char. 354: 1 --> 0

Node 100 :

Char. 3: 0 --> 1

Char. 31: 1 --> 0

Char. 34: 0 --> 1

Char. 54: 0 --> 1

Char. 58: 0 --> 1

Char. 61: 0 --> 1

Char. 67: 0 --> 2

Char. 118: 0 --> 1

Char. 180: 1 --> 0

Char. 184: 0 --> 1

Char. 189: 0 --> 1

Char. 194: 2 --> 1

Char. 207: 1 --> 0

Char. 209: 1 --> 0

Char. 210: 1 --> 0

Char. 268: 1 --> 0

Node 101 :

Char. 35: 0 --> 1

Char. 77: 0 --> 1

Char. 85: 0 --> 1

Char. 90: 1 --> 0

Char. 115: 0 --> 1

Char. 136: 0 --> 1

Char. 166: 0 --> 1

Char. 190: 0 --> 1

Char. 238: 1 --> 0

Char. 241: 0 --> 1

Char. 244: 0 --> 1

Char. 346: 1 --> 0

Node 102 :

Char. 122: 0 --> 1

Char. 226: 1 --> 2

Node 103 :

Char. 50: 0 --> 1

Char. 95: 1 --> 0

Char. 209: 0 --> 1

Char. 210: 0 --> 1

Node 104 :

Char. 170: 0 --> 1

Node 105 :

Char. 60: 0 --> 1

Char. 196: 3 --> 4

Node 106 :

Char. 30: 0 --> 1

Char. 63: 0 --> 1

Char. 80: 0 --> 1

Char. 82: 0 --> 1

Char. 98: 0 --> 1

Char. 104: 0 --> 1

Char. 106: 0 --> 1

Char. 112: 0 --> 1

Char. 114: 0 --> 2

Char. 116: 0 --> 1

Char. 127: 0 --> 1

Char. 138: 0 --> 1

Char. 139: 0 --> 1

Char. 144: 0 --> 1

Char. 178: 0 --> 1

Char. 183: 0 --> 1

Char. 187: 0 --> 1

Char. 211: 0 --> 1

Node 107 :

Char. 8: 0 --> 1

Char. 12: 0 --> 2

Char. 26: 1 --> 0

Char. 31: 1 --> 2

Char. 164: 0 --> 1

Node 108 :

Char. 34: 0 --> 1

Char. 56: 0 --> 1

Char. 58: 0 --> 1

Char. 69: 0 --> 1

Char. 169: 0 --> 1

Node 109 :

Char. 24: 0 --> 1

Char. 27: 0 --> 1

Char. 40: 1 --> 2

Char. 54: 0 --> 1

Char. 101: 1 --> 0

Char. 116: 1 --> 0

Char. 188: 0 --> 1

Node 110 :

Char. 34: 0 --> 1

Char. 74: 0 --> 1

Char. 185: 0 --> 1

Node 111 :

Char. 42: 0 --> 1

Char. 313: 0 --> 1

Char. 320: 1 --> 0

Char. 340: 0 --> 1

Char. 376: 0 --> 1

Node 112 :

Char. 53: 0 --> 1

Char. 102: 1 --> 0

Char. 148: 0 --> 1

Char. 174: 0 --> 1

Char. 259: 0 --> 1

Char. 274: 0 --> 2

Char. 347: 0 --> 1

Char. 357: 0 --> 1

Node 113 :

Char. 1: 0 --> 1

Char. 32: 1 --> 0

Char. 126: 1 --> 0

Char. 151: 0 --> 2

Char. 168: 0 --> 1

Char. 245: 0 --> 1

Char. 253: 1 --> 2

Char. 261: 0 --> 1

Char. 272: 0 --> 1

Char. 305: 0 --> 1

Char. 339: 0 --> 1

Node 114 :

Char. 47: 0 --> 2

Char. 84: 0 --> 1

Char. 228: 0 --> 1

Char. 242: 0 --> 1

Char. 269: 0 --> 1

Char. 309: 0 --> 1

Char. 348: 0 --> 1

Char. 355: 2 --> 0

Char. 370: 0 --> 1

Char. 372: 0 --> 1

Node 115 :

Char. 149: 0 --> 1

Char. 150: 0 --> 1

Node 116 :

Char. 12: 0 --> 1

Char. 29: 0 --> 1

Char. 146: 0 --> 1

Char. 147: 0 --> 1

Char. 164: 0 --> 1

Node 117 :

Char. 15: 0 --> 1

Char. 26: 0 --> 1

Node 118 :

Char. 81: 0 --> 1

Char. 92: 0 --> 1

Char. 101: 1 --> 0

Char. 148: 1 --> 0

Char. 192: 0 --> 1

Char. 240: 0 --> 1

Char. 250: 0 --> 1

Char. 252: 0 --> 1

Char. 260: 0 --> 1

Char. 281: 0 --> 1

Char. 307: 0 --> 1

Char. 341: 0 --> 1

Char. 346: 0 --> 1

Node 119 :

Char. 206: 0 --> 1

Char. 227: 1 --> 2

Char. 251: 0 --> 1

Node 120 :

Char. 58: 0 --> 1

Char. 72: 1 --> 0

Char. 74: 0 --> 1

Char. 76: 0 --> 1

Char. 77: 0 --> 2

Char. 104: 0 --> 1

Char. 109: 0 --> 1

Char. 111: 0 --> 1

Char. 114: 0 --> 1

Char. 115: 0 --> 1

Char. 118: 0 --> 1

Char. 119: 0 --> 1

Char. 163: 1 --> 0

Char. 177: 1 --> 0

Char. 182: 1 --> 0

Char. 194: 0 --> 2

Char. 207: 1 --> 0

Char. 276: 0 --> 1

Char. 277: 0 --> 1

Char. 280: 0 --> 1

Char. 313: 0 --> 1

Char. 355: 2 --> 1

Char. 363: 0 --> 1

Node 121 :

Char. 108: 0 --> 1

Char. 317: 0 --> 1

Node 122 :

Char. 170: 0 --> 1

Char. 195: 0 --> 1

Char. 204: 0 --> 1

Node 123 :

Char. 97: 0 --> 1

Char. 110: 0 --> 1

Node 124 :

Char. 2: 2 --> 1

Char. 102: 0 --> 1

Char. 126: 1 --> 0

Char. 129: 0 --> 1

Char. 178: 0 --> 1

Node 125 :

Char. 33: 0 --> 1

Char. 84: 0 --> 1

Char. 139: 0 --> 1

Node 126 :

Char. 56: 0 --> 1

Char. 164: 0 --> 1

Char. 184: 0 --> 1

Node 127 :

Char. 10: 0 --> 1

Char. 11: 0 --> 1

Char. 17: 0 --> 1

Char. 19: 1 --> 2

Char. 23: 1 --> 2

Char. 24: 0 --> 1

Char. 26: 1 --> 0

Char. 28: 0 --> 1

Char. 32: 1 --> 0

Char. 73: 0 --> 1

Char. 88: 0 --> 1

Char. 156: 1 --> 0

Char. 160: 1 --> 0

Char. 173: 0 --> 1

Node 128 :

Char. 3: 0 --> 1

Char. 12: 0 --> 2

Char. 61: 0 --> 1

Char. 82: 0 --> 1

Node 129 :

Char. 37: 0 --> 2

Char. 93: 2 --> 0

Char. 94: 0 --> 1

Char. 167: 0 --> 1

Node 130 :

Char. 54: 0 --> 1

Char. 169: 0 --> 1
